# Engineered Basic Fibroblast Growth Factor Specifically Bonded with Injectable Extracellular Matrix Hydrogel for the Functional Restoration of Cerebral Ischemia in Rats

**DOI:** 10.34133/bmr.0020

**Published:** 2024-05-02

**Authors:** Chunying Shi, Qi Liu, Feng Sun, Guangyu Zhang, Mingru Deng, Bo Xu, Haicheng Yuan

**Affiliations:** ^1^ The second department of Neurology, Qingdao Central Hospital, University of Health and Rehabilitation Sciences, Qingdao, Shandong Province 266042, China.; ^2^ Department of Human Anatomy, Histology and Embryology, School of Basic Medicine, Qingdao University, Qingdao, Shandong Province 266071, China.; ^3^ Department of Neurology, The Affiliated Hospital of Qingdao University, Qingdao, Shandong Province 266000, China.; ^4^ School of Clinical Medicine, Weifang Medical University, Weifang, Shandong Province 261053, China.; ^5^ Department of Medicine, Qingdao University, Qingdao, Shandong Province 266071, China.

## Abstract

Cerebral ischemia was one of the leading causes of mortality and disability worldwide. Extracellular matrix (ECM) hydrogel held great potential to replace volumetric brain tissue loss following ischemic injury but with limited regenerative effect for functional restoration when implanted alone. In the present study, an engineered basic fibroblast growth factor (EBP-bFGF) was constructed, which fused a specific ECM-binding peptide (EBP peptide) with bFGF. The recombinant EBP-bFGF showed typical binding capacity with ECM without affecting the bioactivity of bFGF both in vitro and in vivo. Furthermore, the EBP-bFGF was used for bioactive modification of ECM hydrogel to repair cerebral ischemia. The combination of EBP-bFGF and ECM hydrogels could realize the sustained release of bFGF in the ischemic brain and improve the regenerative effect of ECM, which protected the survival of neurons, enhanced angiogenesis, and decreased the permeability of blood–brain barrier, ultimately promoted the recovery of motor function. In addition, transcriptome analysis revealed neuregulin-1/AKT pathway involved in this process. Therefore, EBP-bFGF/ECM hydrogel would be a promising therapeutic strategy for cerebral ischemia.

## Introduction

Cerebral ischemia was a common cerebrovascular disease with high mortality and disability rates [1]. Current therapy of cerebral ischemia was used for fast recanalization, including thrombectomy, and recombinant tissue plasminogen activator (rt-PA) but with the limitation of a narrow treatment window and high risk of cerebral hemorrhage [2,3]. In recent years, regenerative therapies have provided potential strategies that improve neurorestoration through neurogenesis, angiogenesis, and blood–brain barrier (BBB) repair [4].

After cerebral ischemia, the interruption of blood flow in the infarcted area caused irreversible damage to the brain that resulted in behavioral and cognitive impairments. The processive loss of neurons and extracellular matrix (ECM) would form a cavity filled with extracellular fluid, which was surrounded by glial scarring to separate the lesion area from the peri-infarct tissue [5,6]. It was reported that the cavity was typically formed 2 weeks after ischemia, with an average size of approximately 45 ml [6]. Currently, there was no effective clinical method to treat this volumetric brain tissue loss.

The ECM formed a 3-dimensional spatial structure for cell adhesion, growth, and mechanotransduction [7]. Because of it retaining the physical, chemical, and biological cues in the regenerative environment, ECM-based scaffolds were revealed to promote tissue regeneration in a series of diseases. For example, ECM-based scaffold such as human dermal acellular matrix and small intestinal submucosal acellular matrix was approved for use in clinical reconstructive surgery [8,9], and noninvasive cardiac ECM hydrogel was also used in clinical trials of myocardial infarction [10]. In the central nervous system (CNS), the ECM-based hydrogels deriving from different organs or tissues showed great potential for cerebral ischemia repair, which could fill in the stroke cavity to promote a structural remodeling, increase the differentiation and neurite outgrowth of neural progenitors, and decrease the infiltration of macrophages [11,12]. Although it served as a promising scaffold for replenishing volumetric brain tissue loss, single ECM-based hydrogel transplantation had relatively limited regenerative effect, and appropriate biological modification with bioactive factors would accelerate the rehabilitation of cerebral ischemia.

In cerebral ischemia, the basic fibroblast growth factor (bFGF) was considered as a multifunctional growth factor, which played an essential role in activating endogenous regeneration. Through different routes such as postischemic administration, intravenous injection, intracerebroventricular infusion, or intranasal penetration, bFGF was revealed to protect neuron survival [13], to activate the neural progenitors, to promote the reconstruction of blood circulation, and to decrease the permeability of BBB [14,15]. However, these traditional treatments were untargeted and unsustainable because of rapid diffusion [16]. Therefore, how to gather bFGF with ECM hydrogel for biological modification and to realize targeted delivery of bFGF was critical for the application in cerebral ischemia.

**Fig. 1.**
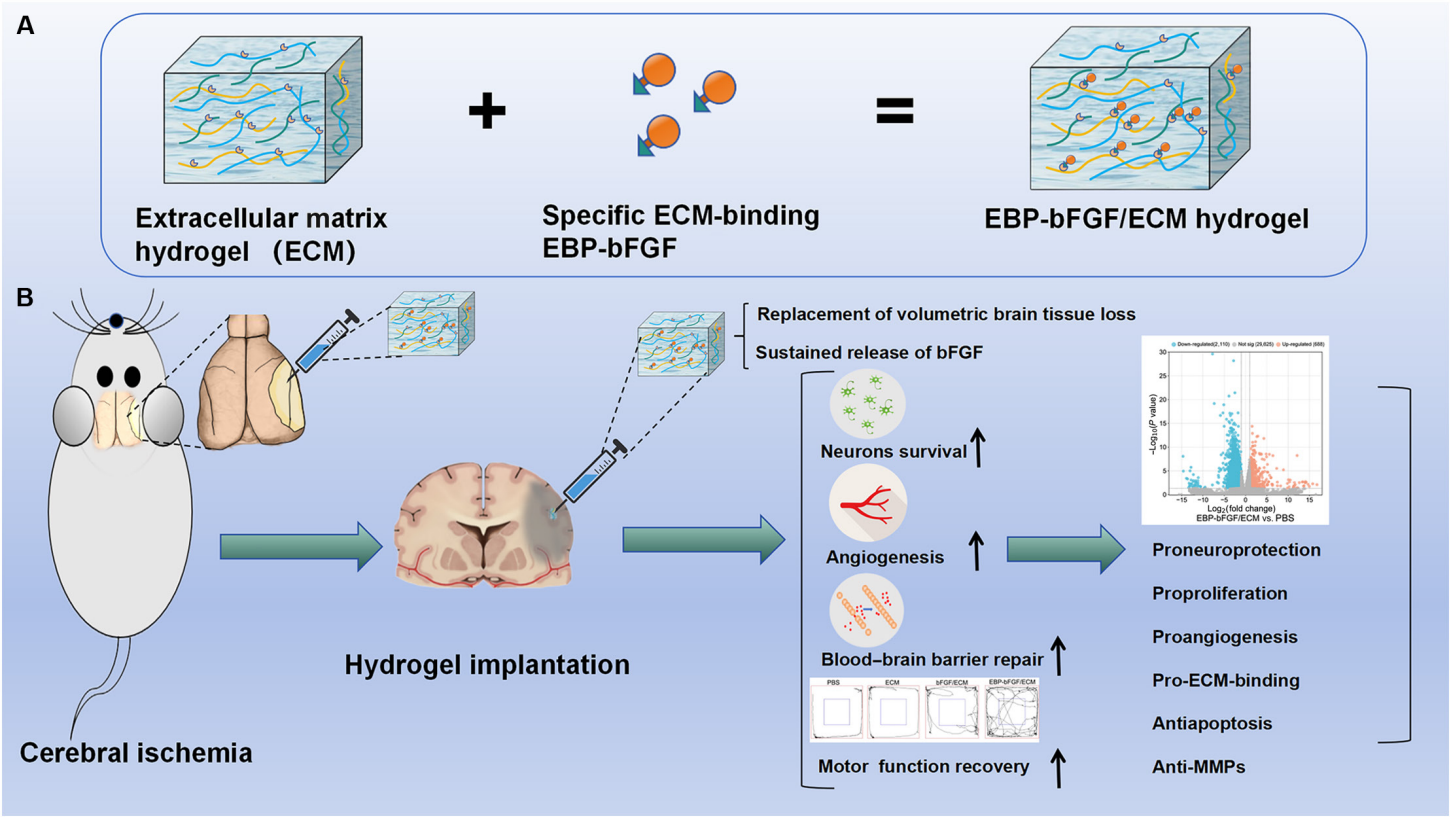
Schematic illustration of engineered EBP-bFGF specifically binding with injectable ECM hydrogel to promote the functional restoration of cerebral ischemia in rats. (A) Preparation of specific ECM-binding EBP-bFGF and construction of injectable EBP-bFGF/ECM hydrogel. (B) Schematic illustration of EBP-bFGF/ECM hydrogel implanted in the ischemic brain, which replaced volumetric brain tissue loss and sustained release of bFGF following ischemic injury to promoted the functional restoration of cerebral ischemia in rats.

Recently, a specific ECM-binding peptide (EBP peptide; RRPKGRGKRRREKQRPTDCHL) deriving from placental growth factor 2 (PlGF-2_123–144_) was demonstrated to bind exceptionally strongly and promiscuously to ECM protein [17]. In present study, the EBP peptide was fused with bFGF to build the recombinant EBP-bFGF protein. The ECM-binding capacity of EBP-bFGF was detected through enzyme-linked immunosorbent assay (ELISA), and the bioactivity of EBP-bFGF was measured through the proliferative assay of human skin fibroblast cells (HSFCs), the survival of pheochromocytoma cells (PC cells) in vitro and subcutaneous embedding in vivo. Then, the middle cerebral artery occlusion and reperfusion (MCAO/R) model of rats were constructed, and the regenerative effect of EBP-bFGF/ECM hydrogel on cerebral ischemia was evaluated by pathological staining and animal motor function measurement. Finally, the potential molecular mechanism of EBP-bFGF/ECM involved in the restoration of cerebral ischemia was explored by RNA sequencing and transcriptome analysis. The key genes and signal pathways were verified, which provided evidence for the validity EBP-bFGF/ECM in the repair of cerebral ischemia (Fig. 1). Therefore, it was hypothesized that EBP-bFGF could typically bond with ECM hydrogel without impacting the bioactivity of both. When EBP-bFGF was used for biological modification of ECM hydrogel, the enhanced regenerative effect would be observed for the repair of cerebral ischemia.

## Materials and Methods

### Preparation of EBP-bFGF and bFGF

The EBP-bFGF was designed by adding the EBP-encoding sequence to bFGF complementary DNA. The gene-encoded EBP-bFGF and native bFGF were inserted into pET28a and transferred into *Escherichia coli* BL21. The *E. coli* BL21 was amplified overnight, and then proteins were induced in 1 mM isopropyl β-d-thiogalactopyranoside at 37 °C for 5 h. After collecting the supernatant obtained from broken *E. coli* BL21, the objective proteins with 6× His tags were purified by nickel chelate chromatography (Amersham Biosciences, Saint Giles Charente, UK). Finally, the EBP-bFGF and bFGF proteins were eluted by gradient imidazole. The recombinant proteins were analyzed for purity and molecular weight size by 15% sodium dodecyl sulfate-polyacrylamide gel electrophoresis (SDS-PAGE) and Western blot.

### Bioactivity assay of EBP-bFGF and bFGF in vitro

The biological activity of EBP-bFGF or bFGF was detected by the proliferative assay of HSFCs. HSFCs at the logarithmic growth stage were cultured in the 48-well plates with a density of 3 × 10^3^ cells per well. Dulbecco’s modified Eagle medium (DMEM; #10564011, Gibco) with 10% fetal bovine serum (FBS; #A5669701, Gibco) was renewed by the DMEM with 2% FBS and the gradient concentration EBP-bFGF or bFGF (0, 312.5, 625, 1,250, and 2,500 pM). Then, HSFCs were incubated in an environment of 5% CO_2_, 95% O_2_, 95% humidity, and 37 °C for 2 d. 3-(4,5-Dimethylthiazole-2-yl)-2,5-diphenyltetrazolium bromide (MTT; 20 μl per well; #M8180, Solarbio) was put in the cell plate for 4 h, and dimethyl sulfoxide (150 μl per well; #D8370, Solarbio) was used to dissolve formazan. Finally, a Universal Microplate Spectrophotometer (CMax Plus) was used to test the cell viability at 492-nm optical density (OD).

### Establishment of oxygen glucose deprivation/reoxygenation model

PC cells (PC12 cells, #CL-0480, Pricella) were cultured into a 48-well plate with 3,000 cells per well in high-glucose DMEM containing 15% horse serum, 2.5% FBS, and 1% penicillin–streptomycin. After the cells adhere and grow to 60%, the complete medium (high-glucose DMEM with 15% horse serum and 2.5% FBS) was replaced with low-glucose DMEM (#SH30021.01, HyClone) with 1% FBS [the oxygen glucose deprivation/reoxygenation (OGD/R) medium] to establish the OGD/R model. The cells were divided into 3 groups with 5 repeated wells and added to the OGD/R medium (200 μl), bFGF (2,500 pM/200 μl), and EBP-bFGF (2,500 pM/200 μl) in each group, respectively. The PC12 cells were placed in a hypoxic incubator (5% CO_2_ and 95% N_2_; Billups-Rothenberg, CA, USA) at 37 °C for 6 h and then followed by the complete medium in the atmosphere of 5% CO_2_, 95% O_2_, 95% humidity, and 37 °C for 18 h. At the same time, the normal cell group was cultured in a 37 °C incubator as the standard for normal cell growth. Finally, cell viability was determined by MTT described previously. The cell survival rate was computed as followed: survival rate (%) = the number of surviving cells / the number of normal cells × 100.

### Preparation of ECM hydrogel

The bladders were collected from 25 to 30 kg of pigs in aseptic environment and put into hyper/hypo tonic NaCl solutions, 0.25% trypsin, 1% SDS, 0.5% Triton-X-100, and sterile water successively with constant stirring. The rest of decellularized ECM was frozen and lyophilized. The microstructure of bladder acellular matrix (BAM) scaffold was observed using scanning electron microscopy (SEM) (model S-2500, Hitachi, Japan), and the biocompatibility of BAM was evaluated by the ingrowth of human bone marrow mesenchymal stem cells (hMSCs) in vitro.

For follow-up experiments, the ECM was ground into powder, and then 1 g of ECM powder was lysed in 0.01 M HCl for 48 h. Finally, the ECM solution was centrifuged and stored in a −80 °C refrigerator for subsequent use. A total of 0.1 N of NaOH and 10× phosphate-buffered saline (PBS) were incorporated into the ECM solution at 4 °C and then put at 37 °C for 30 min to obtain an ECM gel. According to previous studies [18,19], the ECM gel was dissolved in PBS to get a hydrogel. Then, bFGF (1 nmol) and EBP-bFGF (1 nmol) were mixed with ECM gel (1 ml), respectively. After lyophilization (Christ alpha 1-2 LDplus, Osterode, Germany), the microstructure of ECM, bFGF/ECM, and EBP-bFGF/ECM hydrogels was visualized using an SEM operating at an accelerating voltage of 20 kV.

Finally, the degradation of different ECM hydrogels was also evaluated. bFGF (1 nmol) and EBP-bFGF (1 nmol) were mixed with ECM gel (1 ml), respectively, using a 3-way tube and balanced for 30 min at 4 °C. The 3 group samples of ECM, bFGF/ECM, and EBP-bFGF/ECM were added into the 12-transwell plate (500 μl per insert; #3401, Corning Transwell, Sigma-Aldrich, Germany) and cured for 60 min at 37 °C [20]. Then, PBS was added to the peripheral wells of transwell (1 ml per well) to simulate body fluid and replaced every day. The insert was weighed as *M*, and the insert attached sample was recorded as *M*_*X*._ at 0, 1, 3, 5, 7, 14, and 21 d. Retained hydrogel mass and degradation rate were calculated as follows: retained hydrogel mass = *M_X_* − *M*; the degradation rate (%) = (*M_X_* − *M*) / (*M*_0_ − *M*) × 100.

### The binding assay and release assay of EBP-bFGF with ECM and its major component in vitro

To assess the binding ability of EBP-bFGF to urinary bladder matrix hydrogel and its major component hydrogel (Collagen, Fibrin, and Laminin), 100 μl of each hydrogel was encapsulated in a 96-well plate overnight at 4 °C. EBP-bFGF or bFGF was diluted with a PBS solution containing 0.5% bovine serum albumin into a series of concentrations (0, 1.25, 2.5, 3.75, 5, and 10 μM). After discarding and washing the excess ECM, the 2 groups of EBP-bFGF and bFGF (100 μl per well) were added to the 96-well plate at 37 °C for 2 h, respectively (*n* = 5). After washing the plate 3 times with PBS, an anti-bFGF monoclonal antibody (#ab92337, Abcam; 1:500) was used to react with bFGF at 37 °C for 2 h. Then, the plate was incubated with the anti-rabbit immunoglobulin G (IgG) alkaline phosphatase (#A3687, Sigma-Aldrich; 1:20,000) for 1 h. Finally, *P*-nitrophenyl phosphate (2 mg/ml,100 μl per well; #34045, Thermo Scientific) was reacted to color-developing fluid at 37 °C for 10 min and 0.2 M NaOH (100 μl per well) to terminate the experiment. The result was measured using with the Universal Microplate Spectrophotometer at 405-nm OD.

To test the ability to release bFGF, EBP-bFGF and bFGF containing the same amount of bFGF (1 nmol per well) were thoroughly mixed with ECM hydrogel with a 3-way tube and added into a 96-well plate (150 μl per well) to balance for 30 min. PBS (100 μl per well) was added above the hydrogel and the concentration of bFGF in the supernatant was tested at 30 min, 1, 3, 5, and 7 d using the bFGF ELISA kit (#EK0342, BOSTER).

### Animal experiments

The National Institutes of Health “Guide for the Care and Use of Laboratory Animals” (NIH publication 23-80, revised 2011) was used to guide all experiments, and experiment procedure was supported by the Animal Care and Use Committee of Qingdao University (Approval No. QDU-AEC-2023365). Adult male Sprague–Dawley rats weighing 250 to 280 g were purchased from Jinan Pengyue Experimental Animal Breeding Co. Ltd. and fed in a standard environment of free access to food and water, 40% to 60% relative humidity, 25 °C, and a 12-h/12-h light/dark cycle.

#### Subcutaneous embedding

Sterile collagen sponges were cut into 1-cm^2^ size and infiltrated with PBS (100 μl) as the control, bFGF (0.5 nmol/100 μl), and EBP-bFGF (0.5 nmol/100 μl), respectively. A full skin incision of 2 cm was made in the back of anesthetized rats, where sterile collagen sponges were randomly inserted. After 6 and 12 h of sponge implantation, the sponge was removed and added to radioimmunoprecipitation assay lysis solution for grinding using a tissue grinder. Then, after grinding, the mixed liquid was centrifuged to obtain the supernatant, and the remaining bFGF content in the sponge was detected using an ELISA reagent kit (#EK0342, BOSTER).

As described above, sterile sponges containing PBS (100 μl), bFGF (0.5 nmol/100 μl), and EBP-bFGF (0.5 nmol/100 μl) were implanted into the back of rats, respectively. After 14 d of sponge implantation, the rats were executed, and the dorsally implanted sponges and adjacent tissues were removed for observation. After fixation with 10% formalin, 6-μm paraffin sections were prepared. To assess the effects of cell infiltration and proangiogenesis, hematoxylin–eosin (H&E) and α-smooth muscle actin (α-SMA) immunostaining were performed, respectively.

#### MCAO/R model

The MCAO/R model was made following our previous study [21]. Rats were randomly assigned to the PBS group (*n* = 6, as control), the ECM group (*n* = 6), the bFGF/ECM group (*n* = 6), and the EBP-bFGF/ ECM group (*n* = 6). Rats were anesthetized with 2% pentobarbital sodium (50 mg/kg; catalog no. P3761, Sigma-Aldrich), and an incision was cut in the middle of the rat’s neck. The muscles, fascia, nerves, and vessels were sequentially isolated. Then, the right common carotid artery was notched a tiny incision, and a filament (#MSRC37B200PK50, RWD) was intervened from the common carotid artery incision, through the internal carotid artery, to the middle cerebral artery for blocking blood flow. After 1.5 h of occlusion, the brain of rat was reperfusion by pulling out the filament. Finally, 25 μl of PBS, ECM, bFGF/ECM (0.5 nmol), and EBP-bFGF/ECM (0.5 nmol) were injected into the right cerebral cortex at the rate of 2 μl/min within 30 min after the preparation of MCAO model immediately respectively in each group of rats.

#### The release assay of EBP-bFGF with ECM in the ischemic brain

After the cerebral ischemia animal models were constructed, EBP-bFGF/ECM (0.75 nmol) and bFGF/ECM (0.75 nmol) were injected into the brains of Sprague–Dawley rats with cerebral infarctions as described above, respectively. After 3 and 6 h, the ischemic brain tissue and serum were obtained for bFGF content assay by human bFGF ELISA kit (#EK0342, BOSTER) and Western blot.

### Animal behavior measurement

#### Open-field test

At 7 d after reperfusion, rats were subjected to the open-field test. The open field was 100 cm × 100 cm, made of black plastic. The area was divided into 2 regions: central areas (percentage of 25% of total area) and peripheral areas (percentage of 75% of total area). The SMART 3.0 system (Panlab, Spain) was used to record the trails for 5 min. After each experiment, the open-field equipment was cleaned and disinfected to avoid the effects of odors on the experiment.

#### Rotarod test

The rat’s motor coordination was tested by the rotarod test. All rats were trained for 3 d on the rotarod apparatus to ensure that rats learn to use the apparatus. Rats persisted on the rotating bar over 180 s were included in the experiment. The test was conducted 7 d after reperfusion in rats. The data of the speed and the time were collected when the rats fell. Rats were subjected to 3 repeated experiments at 30-min intervals.

#### Claw strength test

The claw strength test was used to evaluate the rat muscle strength and conducted 7 d after reperfusion in rats. The installation (BIO-GS3, Bioseb, Italy), composed of sensors connected to a grid, was used to measure the claw force of rats. The rat was placed on the grid, and then the tail of the rat was dragged horizontally to record the amount of force when the rat was off the grid. Rats were subjected to 3 repeated experiments at 30-min intervals.

### Histological analysis

After anesthesia, rats were injected with 4% paraformaldehyde to perfuse their brains. When the blood was washed out, the brains of rats were taken out and immersed in 4% paraformaldehyde for 48 h for fixation. Then, the samples were dehydrated with 50%, 70%, 90%, and 95% alcohol in sequence, followed by transparency and penetration, and finally placed in the mold for paraffin embedding. To facilitate subsequent staining of slices, the brain sample was cut into a thickness of 5 μm. The morphological changes were evaluated by H&E staining in cerebral infarction areas. Then, we performed fluorescence staining and listed the primary antibodies as follow: anti-neuronal nucleus (NeuN) rabbit polyclonal antibody (#A0951, ABclonal; 1:100), anti-beta III tubulin antibody (Tuj1; #ab18207, Abcam; 1:300), anti-α-SMA antibody (#ab7817, Abcam; 1:100), anti-Von Willebrand factor antibody (vWF; #ab6994, Abcam; 1:400), anti-ZO1 tight junction protein antibody (ZO-1; #ab221547, Abcam; 1:100).

After paraffin sections were dewaxed, hydrated, and antigen-repaired, the tissues were blocked with 20% FBS for 30 min, and the primary antibody was incubated overnight at 4 °C. Then, the goat anti-rabbit IgG (#ab150080, Abcam; 1:500) and goat anti-mouse IgG (#ab150113, Abcam; 1:500) were added separately as a secondary antibody. Finally, the nucleus was stained by 4′,6-diamidino-2-phenylindole (DAPI; #ab104139, Abcam) for 5 min. Different fields were captured in each slice, and the immunofluorescence staining results were analyzed using ImageJ software. In addition, the terminal deoxynucleotidyl transferase-mediated deoxyuridine triphosphate nick end labeling (TUNEL) apoptosis detection kit (#40307ES20, Yeasen) was used to evaluate the cellular apoptosis. The TUNEL assay result was analyzed using ImageJ software. Five perspectives from each slice were counted.

### Transcriptome sequencing

Transcriptome sequencing of the PBS group, ECM group, and EBP-bFGF/ECM group (*n* = 3) was performed to clarify the effects of EBP-bFGF/ECM on differentially expressed genes in cerebral infarction rats. Rats were sampled 5 d after implantation and sequenced by Sangon Biotech for sequencing. Finally, the figures of the differentially expressed genes were constructed using bioinformatics.

### Western blot

The right infarcted brain tissue was lysed with radioimmunoprecipitation assay buffer. Then, proteins were extracted and assayed using the biquinolinic acid protein assay kit (#WB6501, NCM). Quantified proteins were separated via SDS-PAGE gel in different concentrations (10%, 12.5%, and 15%) and transferred to polyvinylidene fluoride membranes. The membranes were closed by tris-buffered saline with Tween with 5% skimmed milk powder for 2 h at 25 °C and incubated with the following primary antibody for 4 °C overnight: anti-bFGF antibody (#sc74412, SCBT; 1:1,000), anti-neuregulin-1 (Nrg1) antibody (#27455-1-AP, Proteintech; 1:5,000), anti-AKT (#A17909, ABclonal; 1:1,000)/anti-p-AKT (#AP0140, ABclonal; 1:1,000), and anti-β-actin rabbit monoclonal antibody (#AC038, ABclonal; 1:5,000). Finally, the membranes were incubated in the goat anti-rabbit IgG (#bs-0295G-HRP, Bioss; 1:5,000) at 25 °C for 1 h and displayed with an automated chemiluminescence image analysis system (Tanon-5200, China) using the Omin-ECL Femto Light Chemiluminescence Kit (SQ201, EpiZyme, China).

### Statistical analysis

Statistical results were reported as means ± SD using GraphPad Prism 8.0.1 (GraphPad Software, CA, USA). Statistical curve fitting was predicted using Microsoft Excel (Microsoft Inc., Redmond, WA). Each experiment would be conducted at least 3 times, and the results of immune tissue staining were analyzed from at least 5 perspectives using ImageJ software (v.1.52a, MD, USA). Unpaired Student’s *t* test was used for comparisons between 2 groups. One-way analysis of variance followed by Tukey’s post hoc test was used for comparison between multiple groups. *P* < 0.05 was considered to have statistical significance and significant differences were indicated by *P < 0.05, **P < 0.01, ***P < 0.001, and ****P < 0.0001.

## Results

### Preparation and bioactivity assays of EBP-bFGF in vitro

**Fig. 2.**
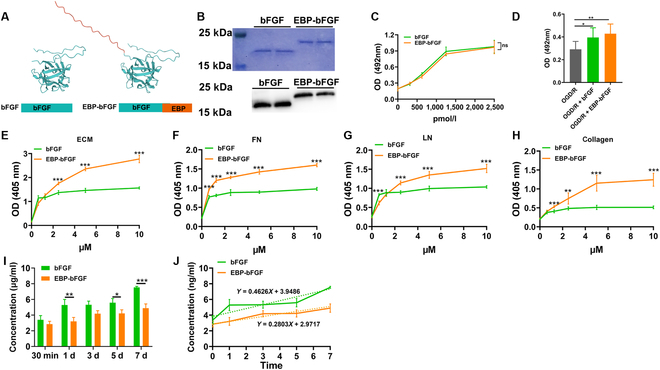
Preparation and properties of the EBP-bFGF and native bFGF. (A) Homologous modeling for predicting the 3-dimensional structure of proteins. In the recombinant EBP-bFGF protein, the EBP short peptide did not affect the spatial conformation of bFGF. (B) The molecular weight of EBP-bFGF and bFGF examined by SDS-PAGE and Western blot. (C) The proliferation activity of bFGF and EBP-bFGF in HSFCs by MTT assay. *n* = 3. (D) The survival activity of PC12 cells in OGD/R by MTT assay. *n* = 5. (E) The binding capacity of bFGF and EBP-bFGF with ECM through ELISA in vitro. *n* = 5. (F) The binding capacity of bFGF and EBP-bFGF with fibronectin (FN) through ELISA in vitro. *n* = 5. (G) The binding capacity of bFGF and EBP-bFGF with laminins (LN) through ELISA in vitro. *n* = 5. (H) The binding capacity of bFGF and EBP-bFGF with collagen through ELISA in vitro. *n* = 5. (I) The release capacity of bFGF and EBP-bFGF with ECM through ELISA in vitro. *n* = 3. (J) The predictive curves for the release of bFGF and EBP-bFGF. Data represent means ± SD. *P < 0.05, **P < 0.01, and ***P < 0.001. ns, not significant.

The recombinant EBP-bFGF was designed, and its 3-dimensional protein modeling was predicted by WeMol. The results showed that the EBP peptide located out of the functional domain of bFGF, and the fusion of EBP peptide did not alter the spatial structure of bFGF (Fig. 2A). Next, the molecular weight was measured by SDS-PAGE and Western blot using the purified native bFGF or EBP-bFGF (Fig. 2B). The result showed that the size of bFGF was about 18.5 kDa and the size of EBP-bFGF was about 23 kDa. Then, the biological activity of bFGF and EBP-bFGF was evaluated in vitro. As shown in Fig. 2C, both bFGF and EBP-bFGF had similar bioactivities to promote the proliferation of HSFCs. As an essential neurotrophic factor, bFGF could protect neurons from the OGD/R model [22]. Subsequently, the effects of EBP-bFGF and bFGF on PC12 cell survival in the OGD/R model were explored. Compared with OGD/R, both bFGF (*P* < 0.05) and EBP-bFGF (*P* < 0.01) promoted the survival of PC12 cells in the OGD/R model (Fig. 2D). These results indicated that the recombinant EBP-bFGF had similar biological activity to bFGF and the addition of the EBP peptide in EBP-bFGF did not affect biological activity of bFGF in vitro.

The BAM was prepared with an average pore size of 100 μm, which had good biocompatibility to promote the growth of hMSCs (Fig. S1A to C), and the ECM hydrogel was prepared as described before [18] (Fig. S1D). Then, the ECM and its main components were prepared to examine the binding ability of EBP-bFGF or bFGF in vitro. The result showed that the bond of EBP-bFGF with ECM was increased in a dose-dependent way, which had significant binding capacity at 2.5 to 10 μM compared to bFGF (*P* < 0.001; Fig. 2E). Similar binding curves of EBP-bFGF to the major components of ECM (Fibrin, Laminin, and Collagen) were also observed (Fig. 2F to H). Moreover, the release of EBP-bFGF from ECM hydrogel was detected. The results showed that significantly less bFGF was released from ECM hydrogel in the EBP-bFGF group compared with the bFGF group at 1 d (*P* < 0.01), 3 d (*P* < 0.05), and 7 d (*P* < 0.001) (Fig. 2I), and the dotted line showed the trend line of bFGF release in which the slope of the EBP-bFGF group was less than bFGF group (Fig. 2J). Finally, we examined whether the binding of bFGF or EBP-bFGF could alter the microstructure of ECM hydrogel. SEM scans of ECM, bFGF/ECM, and EBP-bFGF/ECM hydrogels showed that the average pore size was still about 100 μm, and there were no alterations in both spatial structure and pore size after the combination of engineered bFGFs with ECM hydrogel (Fig. S1E). In addition, the degradation of ECM, bFGF/ECM, and EBP-bFGF hydrogels was also assessed in vitro (Fig. S1F). The degradation curve revealed that all of the 3 groups gradually degraded over time and no significant difference was observed among them at each time point. In general, the average degradation rate of these 3 hydrogels was about 8%, 17%, 33%, 45%, and 61% at 1, 3, 7, 14, and 21 d, respectively, and the total degradative time was estimated about 33 d. Overall, these in vitro results revealed that EBP-bFGF could typically bond with ECM and maintain the sustained release of bFGF, without affecting the microstructure and degradation of ECM hydrogel.

### The binding capacity and bioactivity of EBP-bFGF in vivo

**Fig. 3.**
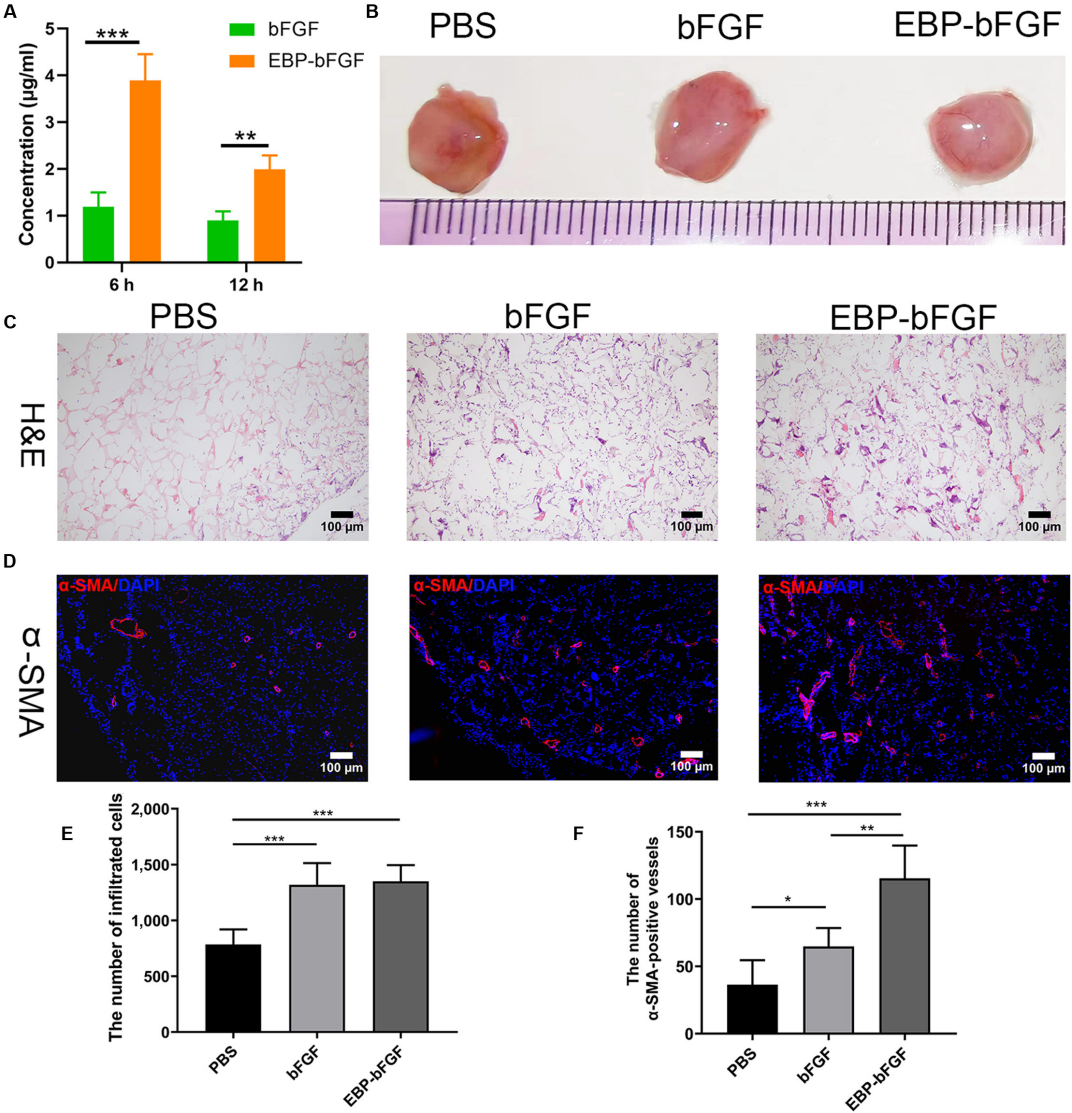
Bioactivity evaluation of EBP-bFGF in subcutaneous implantation in vivo*.* (A) The release capacity of bFGF in the collagen sponge at 6 and 12 h after subcutaneous implantation examined by ELISA assay in vivo. *n* = 3. (B) Gross observation of the sponge at 14 d after subcutaneous implantation in vivo. (C) H&E staining of sponge at 14 d after subcutaneous implantation in vivo. *n* = 5. Scale bars, 100 μm. (D) Immunofluorescence staining of sponge by anti-α-SMA antibody at 14 d after subcutaneous implantation in vivo. *n* = 5. Scale bars, 100 μm. Blue, DAPI; red, α-SMA. (E) Statistical analysis of H&E-positive cell numbers. (F) Statistical analysis of α-SMA-positive vessel numbers. Data represent means ± SD. *P < 0.05, **P < 0.01, and ***P < 0.001.

The binding capacity and biological activity of EBP-bFGF or bFGF in vivo were further evaluated. Since EBP-bFGF could specifically bind with the major component of ECM-collagen in vitro, subcutaneous implantation of collagen sponges loading with EBP-bFGF or bFGF was used to detect the performance of EBP-bFGF in vivo. First, the remnant bFGF in the collagen was detected by ELISA to evaluate the release of EBP-bFGF in vivo. As shown in Fig. 3A, significantly more bFGF was seen in the collagen of the EBP-bFGF group compared with the bFGF group at 6 h (*P* < 0.001) and 12 h (*P* < 0.01) after implantation (Fig. 3A), revealing that EBP-bFGF had specifically binding capacity with major ECM-collagen in vivo. Then, the in vivo bioactivity of EBP-bFGF was assessed by the infiltrated cells and the regenerated blood vessels in collagen sponges. In gross analysis, all implants showed tissue organization with cell infiltration and blood vessel regeneration at 2 weeks after implantation (Fig. 3B). H&E staining of the implants evaluated the cell infiltration. As shown in Fig. 3C, PBS groups (785.0 ± 134.7) had a minimum quantity of cell infiltration. Compared with the PBS group, both the EBP-bFGF group (1,350.0 ± 145.3) and the bFGF group (1,319.0 ± 194.6) exhibited more significant cell infiltration (*P* < 0.001) but with no significant difference between the 2 groups. As an important mitogen, bFGF was an important angiogenic growth factor. Anti-α-SMA immunostaining was used to assess the angiogenesis in the implants. The experiment displayed that the number of blood vessels in the EBP-bFGF group (115.4 ± 24.3) was significantly more numerous than that in the bFGF group (64.8 ± 13.6) and PBS group (36.4 ± 18.2; *P* < 0.001). At the same time, there was a significant difference between the bFGF and PBS groups (*P* < 0.05). Therefore, these results indicated that compared with native bFGF, EBP-bFGF had similar bioactivity to induce cell infiltrations in vivo, and because of EBP-bFGF having a specific binding capacity with collagen sponges, apparent angiogenesis was observed in the EBP-bFGF group.

### The EBP-bFGF/ECM hydrogel decreased the morphological injury in the ischemic brain

**Fig. 4.**
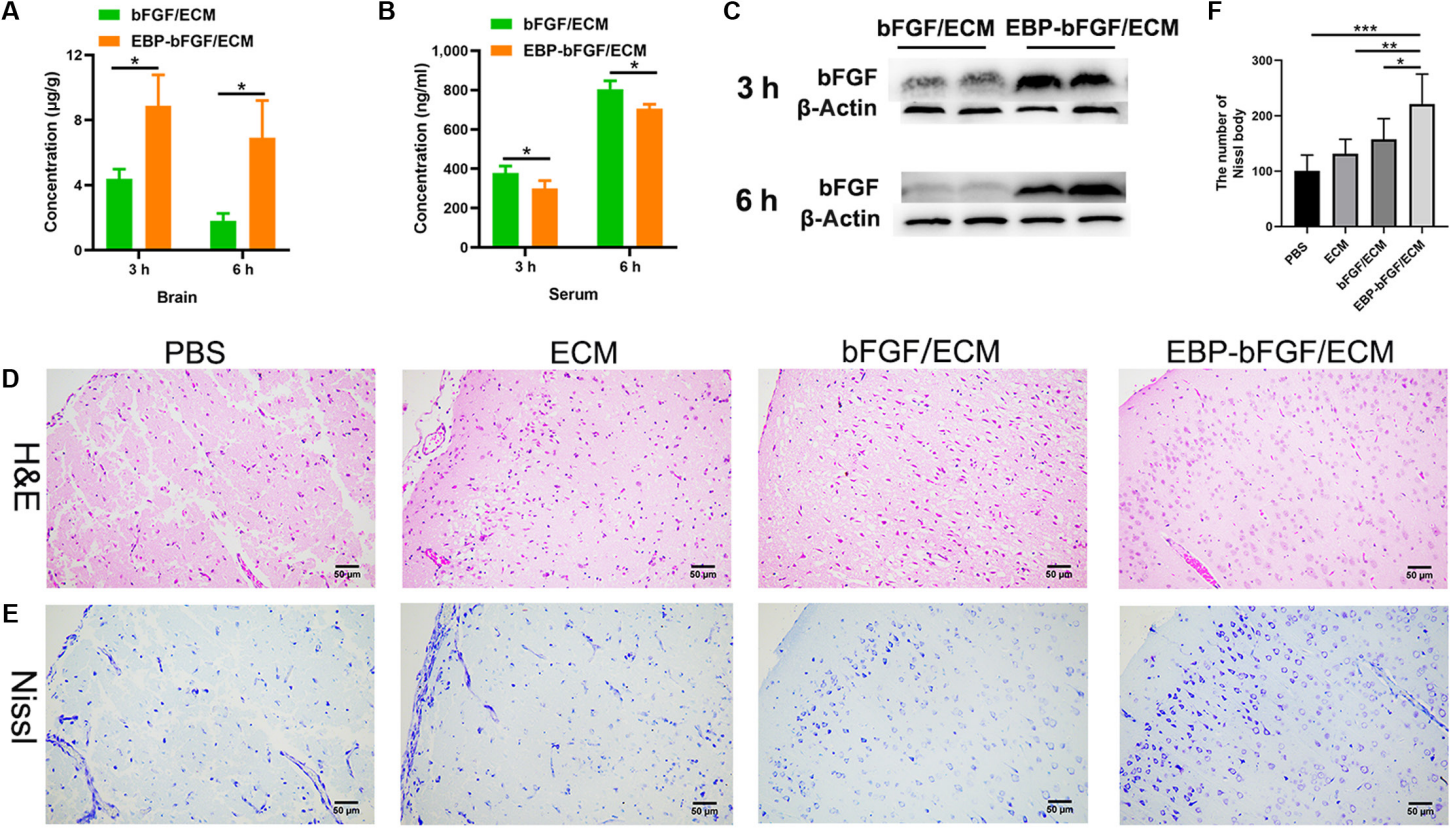
Morphological evaluation of EBP-bFGF/ECM-mediated repair in rats with cerebral ischemia*.* (A) The release capacity of bFGF and EBP-bFGF in the ECM hydrogel at 3 and 6 h in right ischemic brain by ELISA assay. *n* = 3. (B) The release capacity of bFGF and EBP-bFGF in the ECM hydrogel at 3 and 6 h in serum by ELISA assay. *n* = 3. (C) Western blot of bFGF in the right hemisphere at 3 and 6 h. *n* = 3. (D) Histological H&E staining of the ischemic brain at 14 d. *n* = 6. Scale bars, 50 μm. (E) Histological Nissl staining of the ischemic brain at 14 d. *n* = 6. Scale bars, 50 μm. (F) Statistical analysis of Nissl bodies ischemic brain. *n* = 6. Data represent means ± SD. *P < 0.05, **P < 0.01, and ***P < 0.001.

Then, EBP-bFGF or bFGF loading in ECM hydrogel was injected into the ischemic brain in rats of the MCAO model. First, the release of bFGF in brain tissue or serum was detected at 3 and 6 h after administration (Fig. 4A). ELISA assays revealed that the bFGF content in the ischemic brain of the EBP-bFGF/ECM group (8.8 ± 1.8 μg/g) was significantly higher than that of the bFGF/ECM group (4.3 ± 0.5 μg/g) at 3 h after injection (*P* < 0.05), while the bFGF content was 6.9 ± 2.2 μg/g in the EBP-bFGF/ECM group, with significant difference with that of bFGF group (1.8 ± 0.4 μg/g) in the ischemic brain at 6 h after injection (*P* < 0.05). Conversely, in serum, the bFGF content in the EBP-bFGF/ECM group (300.1 ± 39.0 and 705.1 ± 22.7 ng/ml) was lower than that of bFGF/ECM group (378.4 ± 35.6 and 804.7 ± 42.4 ng/ml) at 3 h (*P* < 0.05) and 6 h (*P* < 0.05) after injection, respectively (Fig. 4B). Consistent with the content of bFGF in the ischemic brain, the expression of bFGF was also verified by Western blot (Fig. 4C). These results showed that EBP-bFGF/ECM could retain in the ischemic region and realize sustained release of bFGF. At 2 weeks after implantation, the morphological injury was evaluated by H&E staining and Nissl staining. H&E staining revealed that the EBP-bFGF/ECM group exhibited a distributed and relatively compact arrangement of tissue morphology, while significant neuron loss, shrinkage, and loose arrangement were observed in the PBS group. Moreover, the morphological injury of the ECM group and the bFGF/ECM group lay in between the EBP-bFGF/ECM group and the PBS group (Fig. 4D). In addition, Nissl staining provided more direct evidence for the evaluation of neuron damage. The results showed that in the EBP-bFGF/ECM group, the arrangement of Nissl bodies was regularly and orderly distributed, which had significantly more Nissl bodies (221.3 ± 54.1) compared to the bFGF/ECM group (157.7 ± 37.2), ECM group (131.8 ± 25.8), and PBS group (100.5 ± 28.6) (Fig. 4E and F). Taken together, these findings demonstrated that EBP-bFGF/ECM could decrease the morphological damage of the ischemic brain and exert neuroprotective effects on neurons.

### The EBP-bFGF/ECM hydrogel protected the neurons after stroke

**Fig. 5.**
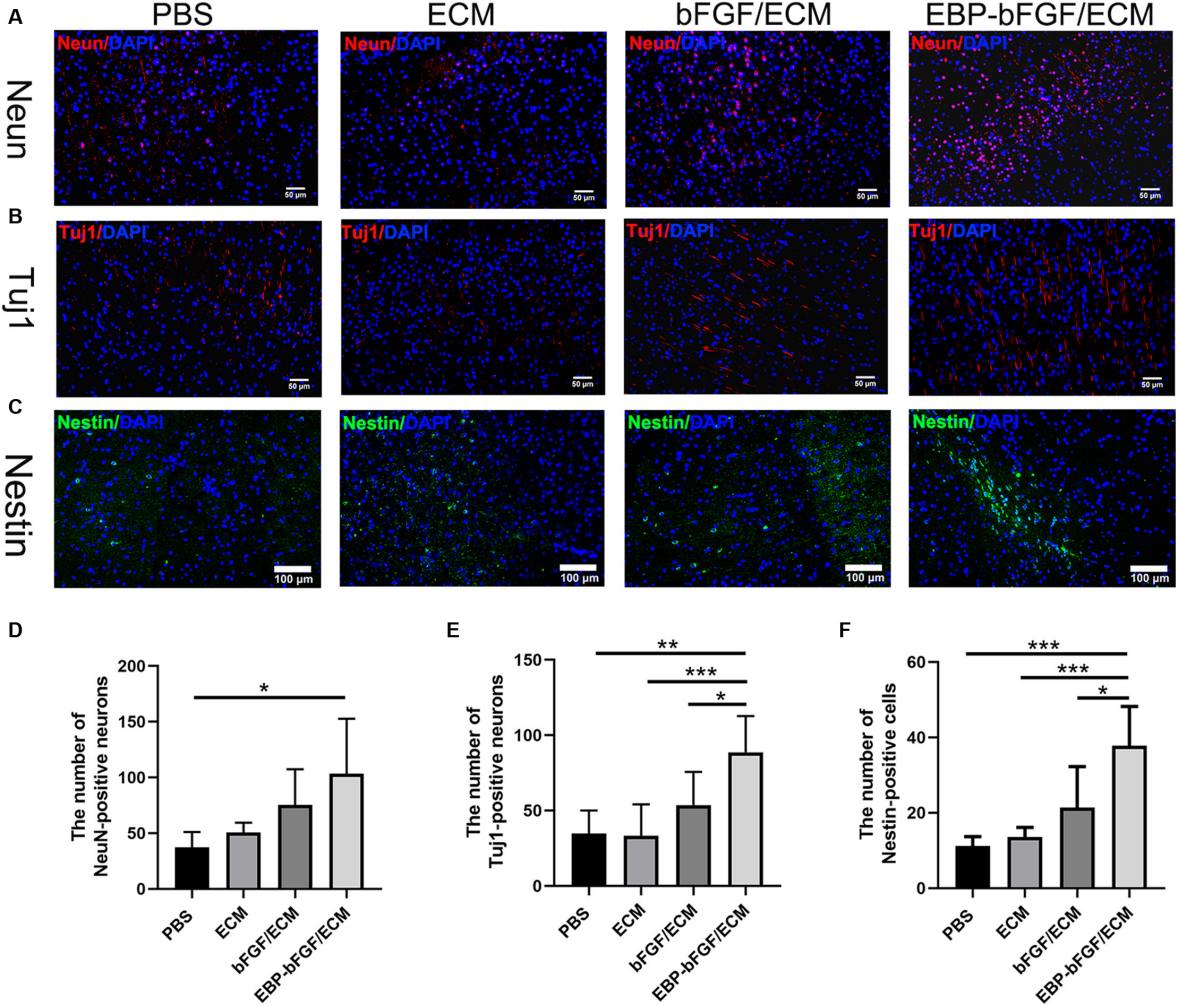
Neuroprotective evaluation of EBP-bFGF/ECM-mediated repair in rats with cerebral ischemia. (A) Immunofluorescence staining of neurons in the ischemic brain. Scale bars, 50 μm. Blue, DAPI; red, Neun. (B) Immunofluorescence staining of axons in the ischemic brain. Scale bars, 50 μm. Blue, DAPI; red, Tuj1. (C) Immunofluorescence staining of neural stem cells in the ischemic brain. Scale bars, 100 μm. Blue, DAPI; green, Nestin. (D) Statistical analysis of NeuN^+^ neurons. *n* = 5. (E) Statistical analysis of Tuj1^+^ neurons. *n* = 6. (F) Statistical analysis of Nestin^+^ neural stem cells. *n* = 5. Data represent means ± SD. *P < 0.05, **P < 0.01, and ***P < 0.001.

The neuroprotection of EBP-bFGF/ECM hydrogel was explored, and antineuron-specific NeuN and anti-Tuj1 were used to evaluate the outcomes of neurons. Anti-NeuN antibody immunostaining was conducted to label neurons, and the results showed that the EBP-bFGF/ECM group protected the survival of neurons, which had significantly more NeuN positive neurons (103.6 ± 49.1) than that of bFGF/ECM group (75.4 ± 32.0), ECM group (50.8 ± 8.5), and PBS group (37.4 ± 13.7) (*P* < 0.05; Fig. 5A and D). Anti-Tuj1 was used to label axons, and the number of axons in the EBP-bFGF/ECM group (88.6 ± 23.9) was significantly more numerous than that in bFGF/ECM group (53.6 ± 22.0; *P* < 0.05), ECM group (33.1 ± 21.0; *P* < 0.001), and PBS group (34.8 ± 15.2; *P* < 0.01) (Fig. 5B and E), which was consistent with the immunostaining of NeuN. In previous studies, bFGF was demonstrated to activate neural stem cells or neural progenitor cells (NPCs) and promoted their differentiation toward neurons in vitro and in vivo [19]. Anti-Nestin antibody was used to identify NPCs, and the number of Nestin-positive cells in the EBP-bFGF/ECM group (37.8 ± 10.4) was significantly higher than that in bFGF/ECM group (21.4 ± 10.8; *P* < 0.05), ECM group (13.6 ± 2.5; *P* < 0.001), and PBS group (11.2 ± 2.4; *P* < 0.001) (Fig. 5C and F). Combined with the neuron immunostaining, it was reasonably speculated that EBP-bFGF/ECM had significant neuroprotective effects and might also be involved in activating NPCs, further facilitating the regeneration and repair of cerebral ischemia.

### The EBP-bFGF/ECM hydrogel promoted angiogenesis and repaired BBB after stroke

**Fig. 6.**
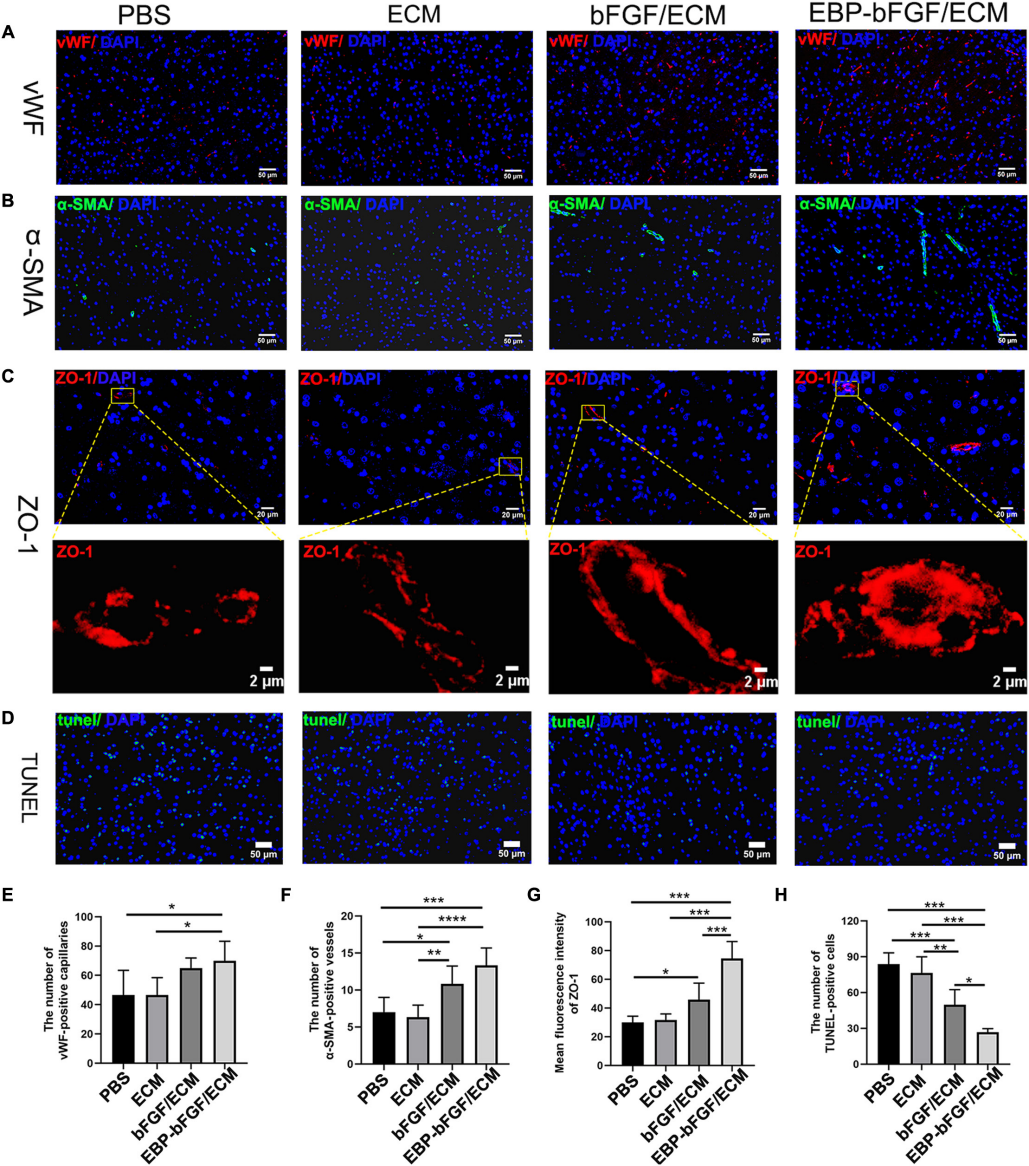
Angiogenesis, BBB repair, and antiapoptotic evaluation of EBP-bFGF/ECM-mediated repair in rats with cerebral ischemia. (A) Immunofluorescence staining of capillaries in the ischemic brain. Scale bars, 50 μm. Blue, DAPI; red, vWF. (B) Immunofluorescence staining of vessels in the ischemic brain. Scale bars, 50 μm. Blue, DAPI; green, α-SMA. (C) Immunofluorescence staining of tight junction proteins in the BBB in the ischemic brain. Scale bars, 20 μm (above) and 2 μm (below). Blue, DAPI; red, ZO-1. (D) TUNEL staining of apoptotic cells in the ischemic brain. Scale bars, 50 μm. Blue, DAPI; green, TUNEL. (E) Statistical analysis of capillaries. *n* = 6. (F) Statistical analysis of α-SMA^+^ vessels. *n* = 6. (G) Statistical analysis of mean fluorescence intensity of ZO-1. *n* = 6. (H) Statistical analysis of TUNEL^+^ cells. *n* = 5. Data represent means ± SD. *P < 0.05, **P < 0.01, ***P < 0.001, and ****P < 0.0001.

Besides neurotrophic factors, bFGF was also a typical inducer of angiogenesis; rapid blood flow restoration was necessary for rehabilitation. As shown in Fig. 6A and E, immunofluorescence staining of anti-vWF was used to identify capillaries. The results showed that more capillaries were detected in the EBP-bFGF/ECM group (70.0 ± 13.3) compared to the ECM group (46.5 ± 11.9; *P* < 0.05) and PBS group (46.5 ± 16.9; *P* < 0.05), with a significant difference. Blood vessels were labeled with anti-α-SMA, and the results showed that the EBP-bFGF/ECM group effectively promoted blood vessel formation. In addition, more α-SMA-positive blood vessels observed in the EBP-bFGF/ECM group (13.3 ± 2.3) than in the bFGF/ECM group (10.8 ± 2.4), ECM group (6.3 ± 1.6; *P* < 0.0001) and PBS group (7.0 ± 2.0; *P* < 0.001) (Fig. 6B and F). It was reported that bFGF could regulate junction proteins of the microvascular barrier and decrease the permeability of BBB or blood spine cord barrier in the brain or spine cord, respectively [15,23,24].The major tight junction protein ZO-1 was detected to measure the effect of bFGF on the BBB of the ischemic brain. The results showed that the mean fluorescence intensity of ZO-1 in the EBP-bFGF/ECM group (74.5 ± 11.7) was typically higher than that in the bFGF/ECM group (45.7 ± 11.5; *P* < 0.001), ECM group (31.6 ± 4.2; *P* < 0.001), and PBS group (30.0 ± 4.2; *P* < 0.001), which the tight junction was more compact than other 3 groups (Fig. 6C and G). Finally, the antiapoptotic ability of ECM and EBP-bFGF was further validated in rats at 14 d of cerebral infarction. As shown in Fig. 6D and H, the EBP-bFGF/ECM group (26.8 ± 2.9) had a significant antiapoptotic ability compared to the other 3 groups: the PBS group (83.8 ± 9.4; *P* < 0.001), the ECM group (76.4 ± 13.5; *P* < 0.001), and the bFGF/ECM group (49.8 ± 12.7; *P* < 0.05).

### The EBP-bFGF/ECM hydrogel promoted the recovery of motor function in cerebral ischemia of rats

**Fig. 7.**
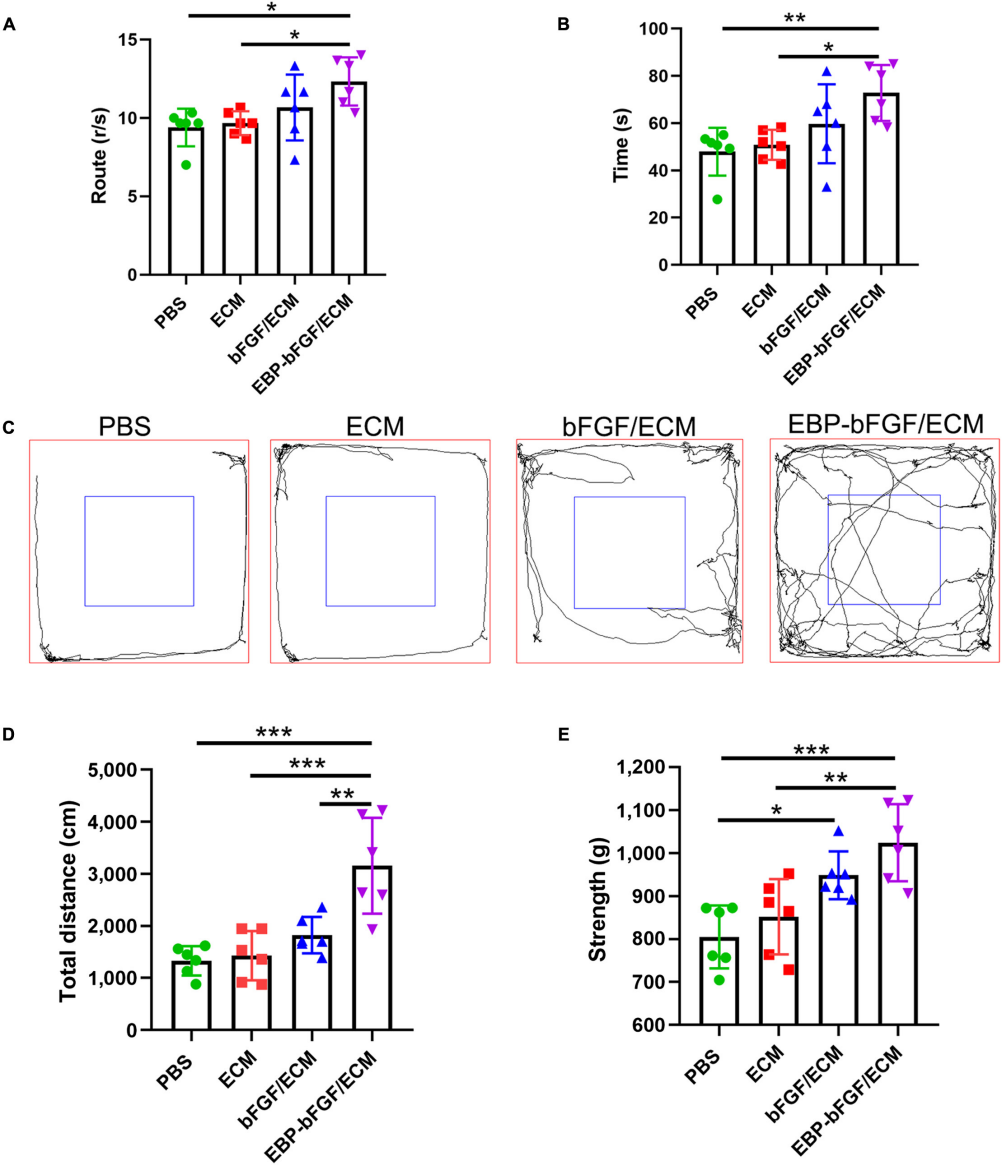
Behavioral test of EBP-bFGF/ECM-mediated repair in rats with cerebral ischemia. (A) Rotarod test on the rotarod apparatus. Statistical analysis of the speed during the rotarod test. *n* = 6. (B) Rotarod test. Statistical analysis of the time during the rotarod test. *n* = 6. (C) The track of the rats during the open-field test was recorded by the SMART 3.0 system. (D) Statistical analysis of the total distance. *n* = 6. (E) The grip strength test by the BIO-GS3 instrument. Statistical analysis of grip strength test. *n* = 6. Data represent means ± SD, *P < 0.05, **P < 0.01, and ***P < 0.001.

Finally, the recovery of the motor system after cerebral infarction in rats was further evaluated using a rotation test, open-field test, and claw strength test. The data of the rotation test indicated that 7 d after reperfusion, there was a notable difference in route between the EBP-bFGF/ECM group (12.3 ± 1.5), ECM group (9.6 ± 0.7; *P* < 0.05), and PBS group (9.3 ± 1.2; *P* < 0.05), while there was no significant difference between bFGF/ECM (10.6 ± 2.0), ECM, and PBS groups (Fig. 7A). Similar results were observed rotation test (Fig. 7B). In addition, open-field tests were conducted to assess the motor function of ischemic rats further. At 7 d after surgery, the results of open-field test showed that the motor trajectories of rats in the EBP-bFGF/ECM group (3,155 ± 921.6) were much longer than those in the bFGF/ECM group (1,823 ± 348.8; *P* < 0.01), ECM group (1,430 ± 473.3; *P* < 0.001), and PBS group (1,329 ± 280.4; *P* < 0.001) (Fig. 7C and D), indicating that the EBP-bFGF/ECM group has more sustained motor ability. In addition, muscle strength was detected by claw strength test at 7 d after reperfusion (Fig. 7E). The data revealed that the rats in EBP-bFGF/ECM group (1,024 ± 89.73) showed greater claw force, which was significantly different from that in PBS group (805.1 ± 73.0; *P* < 0.001) and ECM group (852.0 ± 87.8; *P* < 0.01). There was also a notable difference between the bFGF/ECM group (948.5 ± 55.66) and the PBS group (*P* < 0.05). These results indicated that EBP-bFGF/ECM could improve the recovery of motor function in ischemic rats with better coordination ability and higher movement speed.

### Potential molecular mechanism of EBP-bFGF/ECM hydrogel at 5 d after implantation

**Fig. 8.**
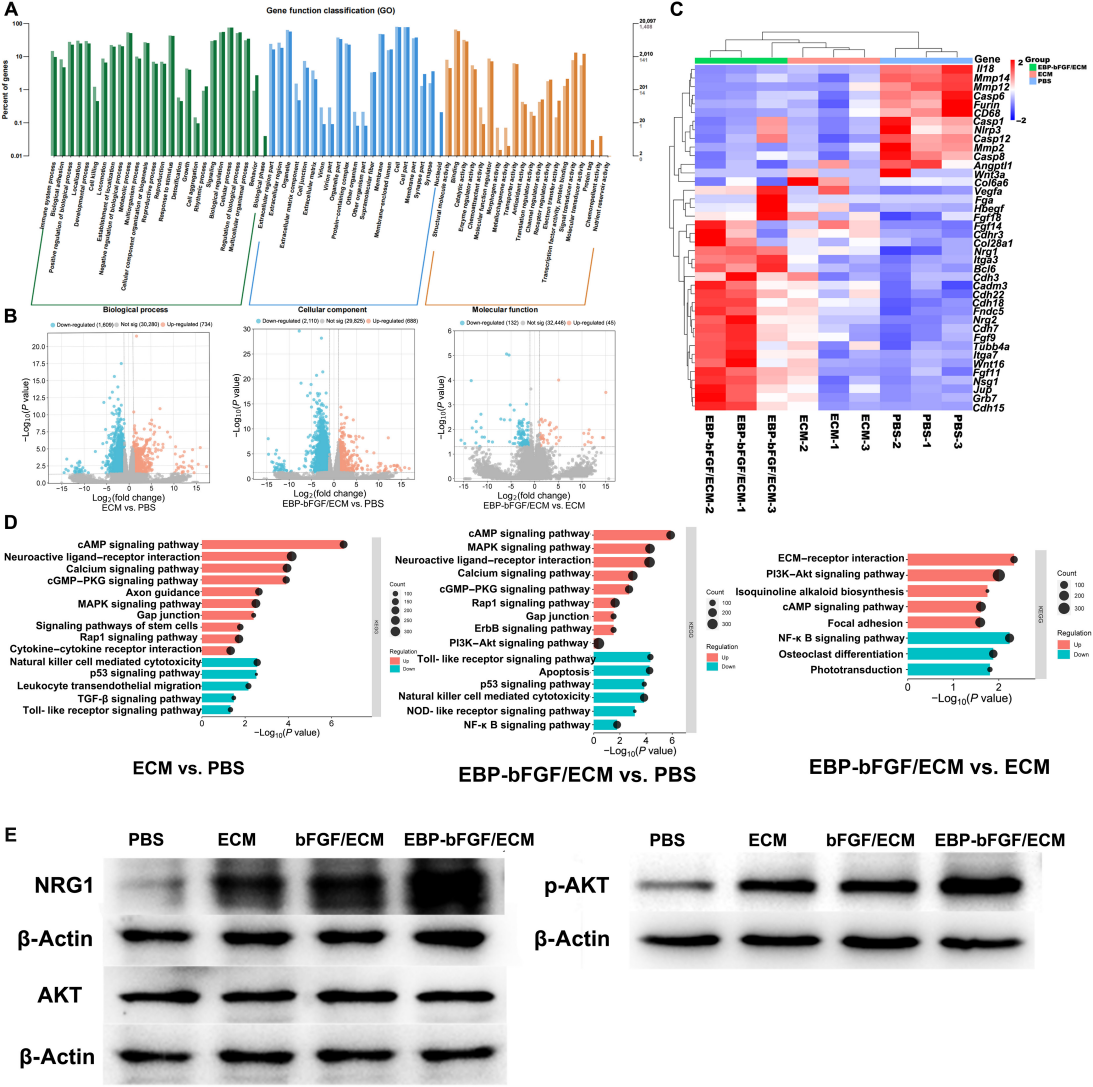
Transcriptome analysis to determine the mechanism of EBP-bFGF/ECM-mediated repair in rats with cerebral ischemia. (A) Gene Ontology (GO) analysis of EBP-bFGF/ECM hydrogel at 5 d after implantation. (B) Differential gene expression of PBS, ECM, and EBP-bFGF/ECM hydrogels at 5 d after implantation. (C) Forty significantly different genes related to regeneration in the ischemic brain. *n* = 3. (D) Kyoto Encyclopedia of Genes and Genomes (KEGG) analysis of PBS, ECM, and EBP-bFGF/ECM hydrogels at 5 d after implantation. cGMP, guanosine 3′,5′-monophosphate; PKG, cGMP-dependent protein kinase; TGF-β, transforming growth factor–β; NOD, nonobese diabetic. (E) Western blot of Nrg1/p-AKT pathway in the right hemisphere at 5 d. *n* = 3.

Transcriptome analysis was used to determine the EBP-bFGF/ECM-mediated repair mechanism in rats with cerebral ischemia. As shown in Fig. 8A, Gene Ontology analysis demonstrated that most genes up-regulated belong to ECM and its component-related cellular component and morphogen activity, as well as metallochaperone-activity-related molecular function; in contrast, most of the down-regulated genes belong to signal-transducer-activity- and molecular-transducer-activity-related molecular functions. EBP-bFGF/ECM group up-regulated 45 genes and down-regulated 132 genes compared to ECM group and up-regulated 688 genes and down-regulated 2,010 genes in comparison to PBS group (Fig. 8B). Differentially expressed genes were identified by analyzing fragments per kilobase of exon model per million fragments mapped data (Fig. 8C). Compared with EBP-bFGF/ECM group and ECM group, a series of an inflammatory gene such as Interleukin18 (*IL18*), *CD68*, and nucleotide-binding domain, leucine-rich repeat, and pyrin domain-containing protein 3 (*Nlrp3*); apoptosis genes such as *caspase 6*, *caspase 1*, *caspase12*, and *caspase 8*; and matrix metalloproteinase (MMP) family including *MMP12*, *MMP14*, and *MMP2* were typically up-regulated in PBS group, which indicated the potential protective function of ECM hydrogels. On the contrary, genes related to neurotrophic factors and proangiogenesis such as *NRG1*, *NRG2*, *FGF9*, and *FGF11*, neuron-specific gene family member 1 (*NSG1*) were significantly up-regulated in EBP-bFGF/ECM group compared with the ECM and PBS groups, which was consistent with the neuroprotective and angiogenic effects of EBP-bFGF/ECM describe above. Furthermore, ECM-related proteins such as integrin family (*Itga3* and *Itga7*), cadherin family (*Cdh3*, *Cdh18*, *Cdh7*, *Cdh22*, and *Cdh15*), cell adhesion molecule 3 (*CADM3*), Tubulin beta-4a (*TUBB4*), junction plakoglobin (*JUP*) were also up-regulated in EBP-bFGF/ECM group compared with the ECM and PBS groups. The genes were tightly related to the interaction of EBP peptides and ECM. Then, the Kyoto Encyclopedia of Genes and Genomes database was used to identify signaling pathways that regulated neural repair in the EBP-bFGF/ECM group. As shown in Fig. 8D, the EBP-bFGF/ECM and ECM groups substantially activated the adenosine 3′,5′-monophosphate (cAMP) signaling pathway, neuroactive ligand–receptor interaction, and mitogen-activated protein kinase (MAPK) signaling pathway compared with the PBS group, and the EBP-bFGF/ECM group mainly up-regulated phosphatidylinositol 3-kinase (PI3K)–AKT signaling pathway, ECM receptor interaction, and focal adhesion compared with ECM group. These results were consistent with previous studies. As the neuroprotective factor, *NGR1* was demonstrated to regulate downstream pathways of the PI3K–AKT signaling pathway to repair cerebral ischemia [25,26]. Finally, the main differentially expressed gene *NRG1* and AKT pathway was validated by Western blot, and the results showed that the expression of *NRG1* was evidently higher in the EBP-bFGF/ECM group compared with the bFGF/ECM, ECM, and PBS groups, which was accompanied with the obvious activation of p-AKT (Fig. 8E).

## Discussion

Stroke, with a high incidence rate, high mortality, and high disability rate, was the leading cause of death, which seriously threatened human life and health. In recent years, it was reported that the absolute number of strokes had increased by 70.0%, stroke deaths had risen by 43.0%, and the age distribution of strokes had shown a younger trend [27]. The current clinical treatment mainly focuses on rt-PA thrombolysis [28] and mechanical thrombectomy [29], which aim at rescuing blood flow in the ischemic penumbra. However, the therapeutic time window of thrombolysis was narrow. Because of these limitations, it was urgent to explore novel treatments for the therapy of stroke.

ECM-based scaffolds have been shown to promote the reconstruction of injured tissues and facilitate functional tissue restoration [30]. ECM hydrogels deriving from different sources, such as the brain, bladder, and spine cord, were compared in cerebral injury. These ECM hydrogels were revealed to promote the remodeling of injured tissue, which enhanced the proliferation and migration of neural progenitors or neurons and facilitated the polarization of macrophages from proinflammatory M1 toward prorepair M2 phenotype in vitro [31]. Among these ECM hydrogels, BAM-derived ECM hydrogel was most attractive. A series of previous studies demonstrated the most significant increase in differentiation and neurite outgrowth of neural progenitors with BAM-derived ECM rather than CNS-derived ECM in vitro. That was because intrinsic inhibitors in axon sprouting might be retained in CNS-derived ECM, and the yield of CNS-derived ECM was much lower than BAM-derived ECM [5,6]. As a result, BAM-derived ECM hydrogel was explored for the repair of cerebral ischemia by in situ administration and was revealed to fill in the stroke cavity, replacing the volumetric brain tissue loss, which promoted structural remodeling and partially induced neural tissue restoration. In addition, the regenerative effects of different concentrations of BAM-derived ECM hydrogel (3, 4, and 8 mg/ml) were also compared, and the hydrogel (4 mg/ml) had the most favorable characteristics for brain regeneration, which efficiently degraded and supported better neuron, astrocyte, and endothelial cells infiltration [32]. In the present study, BAM-derived ECM hydrogel (4 mg/ml) was used.

bFGF was an important neurotrophic factor for neurogenesis, neuroprotection, and angiogenesis. In the treatment of cerebral ischemia, targeting delivery of bFGF was highlighted to increase local concentration, and different methods were investigated. The intranasal use of bFGF nanoliposomes was explored for functional recovery in rodent cerebral ischemia model [13,33]; platelet-membrane-cloaked nanocarriers were used to delivery bFGF that promoted angiogenesis and neurogenesis and neurobehavioral recovery [34]; biodegradable polyglycolic acid sheets releasing bFGF was revealed to promote angiogenesis and activated NPCs in mice [16]. In our previous study, a reconstructed CTGF binding peptide-bFGF (CFBP-bFGF) was designed that could specifically deliver to the ischemic brain through intravenous injection by binding with the up-regulated connective tissue growth factor in injured microenvironment [21].

Here, an ECM-binding EBP-bFGF was designed and prepared, aiming at the biological modification of ECM hydrogel. First, the binding capacity and bioactivity of EBP-bFGF were verified. WeMol homology modeling indicated that the EBP peptide located out of the functional domain of bFGF (Fig. 2A). After the purification of EBP-bFGF or native bFGF (Fig. 2B, C), the bioactivity of both bFGF was detected by the proliferative assays of HSFCs and the survival assay of PC12 cells. Similar proliferative curves were observed in different concentrations of EBP-bFGF and bFGF (Fig. 2E). Both of them had a similar neuroprotective effect on PC12 cells in the OGD/R model (Fig. 2F). These results showed that because of EBP peptide located as an independent functional domain, the EBP peptide did not affect the bioactivity of bFGF. The bonding capacity of EBP-bFGF with ECM hydrogel was further evaluated, and EBP-bFGF had typical binding ability with ECM and its major components, which released much more slowly from the ECM compared with bFGF (Fig. 2E to J). These results were confirmed by subcutaneous embedding animal models in vivo, and the combination of EBP-bFGF and collagen ECM matrix could decrease the release of bFGF in the matrix that promotes cell infiltration and angiogenesis in remodeling tissues effectively (Fig. 3). Therefore, these data suggested that through the superior binding capacity of EBP-bFGF with ECM, EBP-bFGF would be a potent candidate for the bioactive modification of ECM, which also limited the rapid diffusion of bFGF in local injected region to retain effective concentration.

As bFGF was a crucial neurotrophic factor, it played an important role in neuroprotection, neurogenesis, and angiogenesis. Then, the ECM hydrogel loading with EBP-bFGF was implanted into the ischemic brain, and the regenerative effects were further explored. Two weeks after implantation, decreased morphological injury with exhibited well-distributed tissue morphology and regularly arranged Nissl bodies were observed by H&E staining and Nissl staining (Fig. 4). Moreover, the neuroprotective effects were further verified by antineuron-specific NeuN and anti-Tuj1 immunostaining. Consistent with morphological staining, significantly more neurons and axons were observed in EBP-bFGF/ECM hydrogel than in the other 3 groups (Fig. 5A, B, D, and E). Because bFGF could activate endogenous NPCs [35] and EBP-bFGF could retain its effective concentration in the ischemic brain, the number of Nestin-positive NPCs in the EBP-bFGF/ECM group was also statistically higher than that in bFGF/ECM, ECM, and PBS groups (Fig. 5C and F). In addition, angiogenesis was measured, and the results showed that EBP-bFGF/ECM hydrogel promoted the regeneration of blood vessels and formed more tight junctions to decrease the permeability of BBB (Fig. 6). Because EBP-bFGF/ECM hydrogel could mediate neuronal protection, promote angiogenesis, and reduce the permeability of BBB, the improvement of motor function recovery was also observed compared with bFGF/ECM, ECM, and PBS groups (Fig. 7). Compared with other delivery scaffolds, ECM was naturally derived, avoiding the side effects of the foreign carrier, and because of the bridging effect of EBP peptide, EBP-bFGF could realize the sustained release of bFGF from ECM hydrogel that facilitated the morphological and functional recovery of cerebral ischemia.

Although ECM hydrogel was shown to promote the remodeling of the ischemic brain in previous studies, the downstream signaling pathway was still unknown. In the present study, the transcriptome analysis results (Fig. 8) demonstrated that inflammatory genes, apoptosis genes, and MMP genes were typically up-regulated in the PBS group compared with the ECM and EBP-bFGF/ECM groups at 5 d after implantation. In cerebral ischemia, inflammatory, apoptosis, and MMP family genes were closely related. In the acute phase, the activation of neuroinflammatory cascades directly caused apoptosis, the BBB [36], and MMPs had been shown to degrade components of the basal lamina, leading to disruption of the BBB, which contributed to leukocyte infiltration and progressive neuroinflammatory reactions, even triggered underlying cerebral edema, hemorrhage, and brain tissue loss [37]. These results suggested the neuroprotective role of ECM hydrogel. Furthermore, the molecular mechanism of EBP-bFGF/ECM hydrogel was further investigated. A series of regeneration-related genes and signaling pathways, including neurotrophic factors and proangiogenic factors, were significantly up-regulated in EBP-bFGF/ECM hydrogel compared with the ECM and PBS groups. Among them, NRG1 and its downstream AKT pathway were emphasized (Fig. 8C to E). NRG1 was transduced through the receptor tyrosine-protein kinase erythroblastic oncogene B (ErbB) family to regulate downstream pathways PI3K–AKT and PI3K–extracellular-signal-regulated kinase signaling pathway. It had been demonstrated NRG1 signaling involved in NPCs activation [38], neuroprotective effects [39,40], neuroinflammatory modulation [41,42], and angiogenesis [43]. Through Western blot, the expression of NRG1 and AKT, typically activated in the EBP-bFGF/ECM group, was verified compared with the other 2 groups. These results indicated that the sustained release of EBP-bFGF might coordinate the NRG1–AKT signaling pathway to regulate the neural repair. In addition, ECM-binding-related genes such as integrins were also raised in the EBP-bFGF/ECM group compared with the ECM and PBS groups. In a previous study of matrix-bound vascular endothelial growth factor (VEGF), integrins were revealed to facilitate and enhance VEGF receptor 2 (VEGFR2) activation by inducing VEGFR2 clustering [44]. Therefore, we speculated the activation of these ECM-binding-related genes might involve the interaction of ECM and EBP peptides; whether these genes could participate in the activation of the receptor of bFGF was unknown, and further investigations would be required.

The balance between ECM degradation and tissue regeneration was essential for the restoration of injured tissues and the gradual degradation of ECM should match with the infiltration of cells and endogenous regeneration process. In previous studies, Modo’s group [20] investigated the degradation of different concentration of urinary bladder-ECM hydrogel in vitro and in vivo. With the increase in hydrogel concentration, the degradation of ECM hydrogel decelerated in vitro [20]. When these ECM hydrogels were implanted into the stroke cavity in vivo, a 95% volume was availably degraded in low concentrated hydrogels by 90 d, such as 3 and 4 mg/ml, while only 32% of the high concentrated hydrogel (8 mg/ml) was degraded. All hydrogel concentrations promoted the infiltration of neural cells. However, the hydrogels (4 mg/ml) showed an intense invasion of endothelial cells with neovascularization, but no neovascularization emerged with the stiffer hydrogel (8 mg/ml) [5]. Overall, it was accepted that hydrogel (4 mg/ml) was the optimal candidate for comprehensive consideration of ECM degradation and tissue repair in cerebral ischemia. To detect whether the combination of bFGF or EBP-bFGF could impact the microstructure and degradation of ECM hydrogel (4 mg/ml), the SEM and in vitro degradative assay were performed in Fig. S1E and F. The average pore size of bFGF/ECM and EBP-bFGF/ECM hydrogels was also about 100 μm, similar with that of ECM hydrogel that was observed by SEM, and the degradation of bFGF/ECM and EBP-bFGF/ECM hydrogels had also no significance with ECM hydrogel in simulated body fluid environment. These results indicated the specific binding of bFGF or EBP-bFGF with ECM did not impact its microstructure and degradation of the scaffold in vitro. In subcutaneous embedding models in vivo (Fig. 2), as EBP-bFGF/collagen showed better the cell infiltration and angiogenesis than bFGF/collagen and collagen scaffold, the smaller size and more visible tissue remodeling of the implant was observed. As a result, it was reasonably speculated that because EBP-bFGF/ECM hydrogel could accelerate the tissue regeneration in cerebral ischemia animal model, the degradation of EBP-bFGF/ECM hydrogel would be faster than bFGF/ECM hydrogel and ECM hydrogel. In the following studies, the degradation of EBP-bFGF/ECM hydrogel in stroke cavity in vivo will be explored to further validate the relationship between ECM degradation and EBP-bFGF-induced tissue regeneration.

The injectable ECM hydrogel was a promising therapeutic approach for tissue reconstruction, and how to improve its regenerative effect was emphasized in the field of tissue engineering. In present study, EBP-bFGF was designed and constructed that had particular high affinity with ECM without altering the structural or biological properties of ECM itself. It was dedicated for biological modification of ECM that could dramatically improve the regenerative efficacy of both bFGF and ECM hydrogel, providing an effective strategy for the repair of cerebral ischemia to replace volumetric brain tissue loss. However, the regenerative effect of EBP-bFGF/ECM hydrogel was investigated at 14 d after hydrogel implantation, which was a relatively short observation period and was a limitation of present study. In addition, beside the therapy of cerebral ischemia, the application of injectable ECM hydrogels was wide including skin wound healing, myocardial infarction, bone and cartilage defect, etc. Currently, the verification of effectiveness of EBP-bFGF/ECM hydrogel was only selected MCAO animal model that was another limitation of this study.

### Conclusion

In present study, an engineered recombinant EBP-bFGF was constructed, which showed typical binding capacity with ECM without impacting the bioactivity of bFGF. The EBP-bFGF/ECM hydrogel could realize the sustained release of bFGF in the ischemic brain and improve the regenerative effect of ECM, while the survival of neurons, the regeneration of blood vessels, the repair of the BBB, and the recovery of motor function were observed. In addition, the underlying mechanism of EBP-bFGF/ECM was explored by transcriptome analysis and the neuroprotective NRG1/AKT signaling pathway involved in the repair of cerebral ischemia. Further, the route of administration through an image-guided procedure will be explored, appropriate dosage and injection time will be optimized, and long-term observation will be conducted for EBP-bFGF/ECM in the treatment of cerebral ischemia, which will provide definite evidence for further clinical translational applications.

## Ethics Approval and Consent to Participate

All experiment procedures were supported by the Animal Care and Use Committee of Qingdao University (approval no. QDU-AEC-2023365).

## Data Availability

All data required of the paper are included in the paper and/or the Supplementary Materials. The other related data may be requested from the authors.

## References

[1] Diseases GBD, Injuries C. Global burden of 369 diseases and injuries in 204 countries and territories, 1990-2019: A systematic analysis for the global burden of disease study 2019. Lancet. 2020;396(10258):1204–1222. 33069326 10.1016/S0140-6736(20)30925-9 PMC7567026

[2] Raffaele S, Gelosa P, Bonfanti E, Lombardi M, Castiglioni L, Cimino M, Sironi L, Abbracchio MP, Verderio C, Fumagalli M. Microglial vesicles improve post-stroke recovery by preventing immune cell senescence and favoring oligodendrogenesis. Mol Ther. 2021;29(4):1439–1458. 33309882 10.1016/j.ymthe.2020.12.009 PMC8058432

[3] Boese AC, Le QE, Pham D, Hamblin MH, Lee JP. Neural stem cell therapy for subacute and chronic ischemic stroke. Stem Cell Res Ther. 2018;9(1):154. 29895321 10.1186/s13287-018-0913-2 PMC5998588

[4] Wu Y, Sun J, Lin Q, Wang D, Hai J. Sustained release of vascular endothelial growth factor A and basic fibroblast growth factor from nanofiber membranes reduces oxygen/glucose deprivation-induced injury to neurovascular units. Neural Regen Res. 2024;19(4):887–894. 37843225 10.4103/1673-5374.382252 PMC10664103

[5] Ghuman H, Mauney C, Donnelly J, Massensini AR, Badylak SF, Modo M. Biodegradation of ECM hydrogel promotes endogenous brain tissue restoration in a rat model of stroke. Acta Biomater. 2018;80:66–84. 30232030 10.1016/j.actbio.2018.09.020 PMC6217851

[6] Modo M, Badylak SF. A roadmap for promoting endogenous in situ tissue restoration using inductive bioscaffolds after acute brain injury. Brain Res Bull. 2019;150:136–149. 31128250 10.1016/j.brainresbull.2019.05.013 PMC6626582

[7] Potjewyd G, Kellett KAB, Hooper NM. 3D hydrogel models of the neurovascular unit to investigate blood-brain barrier dysfunction. Neuronal Signals. 2021;5(4):NS20210027. 10.1042/NS20210027 PMC8579151 34804595

[8] Mihalecko J, Bohac M, Danisovic L, Koller J, Varga I, Kuniakova M. Acellular dermal matrix in plastic and reconstructive surgery. Physiol Res. 2022;71:S51–S57. 36592440 10.33549/physiolres.935045 PMC9854008

[9] Fujii M, Tanaka R. Porcine small intestinal submucosa alters the biochemical properties of wound healing: A narrative review. Biomedicines. 2022;10(9):2213. 36140314 10.3390/biomedicines10092213 PMC9496019

[10] Hunter JD, Johnson TD, Braden RL, Christman KL. Injectable ECM scaffolds for cardiac repair. Methods Mol Biol. 2022;2485:255–268. 35618911 10.1007/978-1-0716-2261-2_17

[11] Modo M, Ghuman H, Azar R, Krafty R, Badylak SF, Hitchens TK. Mapping the acute time course of immune cell infiltration into an ECM hydrogel in a rat model of stroke using ^19^F MRI. Biomaterials. 2022;282: Article 121386. 35093825 10.1016/j.biomaterials.2022.121386

[12] Damian C, Ghuman H, Mauney C, Azar R, Reinartz J, Badylak SF, Modo M. Post-stroke timing of ECM hydrogel implantation affects biodegradation and tissue restoration. Int J Mol Sci. 2021;22(21):11372. 34768800 10.3390/ijms222111372 PMC8583606

[13] Zhao YZ, Lin M, Lin Q, Yang W, Yu XC, Tian FR, Mao KL, Yang JJ, Lu CT, Wong HL. Intranasal delivery of bFGF with nanoliposomes enhances in vivo neuroprotection and neural injury recovery in a rodent stroke model. J Control Release. 2016;224:165–175. 26774220 10.1016/j.jconrel.2016.01.017

[14] Katoh M. Therapeutics targeting FGF signaling network in human diseases. Trends Pharmacol Sci. 2016;37(12):1081–1096. 27992319 10.1016/j.tips.2016.10.003

[15] Chen P, Tang H, Zhang Q, Xu L, Zhou W, Hu X, Deng Y, Zhang L. Basic fibroblast growth factor (bFGF) protects the blood-brain barrier by binding of FGFR1 and activating the ERK signaling pathway after intra-abdominal hypertension and traumatic brain injury. Med Sci Monit. 2020;26: Article e922009. 32036381 10.12659/MSM.922009 PMC7029819

[16] Ito Y, Oyane A, Yasunaga M, Hirata K, Hirose M, Tsurushima H, Ito Y, Matsumaru Y, Ishikawa E. Induction of angiogenesis and neural progenitor cells by basic fibroblast growth factor-releasing polyglycolic acid sheet following focal cerebral infarction in mice. J Biomed Mater Res A. 2022;110(12):1964–1975. 36183359 10.1002/jbm.a.37434

[17] Martino MM, Briquez PS, Guc E, Tortelli F, Kilarski WW, Metzger S, Rice JJ, Kuhn GA, Muller R, Swartz MA, et al. Growth factors engineered for super-affinity to the extracellular matrix enhance tissue healing. Science. 2014;343(6173):885–888. 24558160 10.1126/science.1247663

[18] Jiang D, Huang J, Shao H, Hu X, Song L, Zhang Y. Characterization of bladder acellular matrix hydrogel with inherent bioactive factors. Mater Sci Eng C Mater Biol Appl. 2017;77:184–189. 28532019 10.1016/j.msec.2017.03.222

[19] Li Y, Yang L, Hu F, Xu J, Ye J, Liu S, Wang L, Zhuo M, Ran B, Zhang H, et al. Novel thermosensitive hydrogel promotes spinal cord repair by regulating mitochondrial function. ACS Appl Mater Interfaces. 2022;14(22):25155–25172. 35618676 10.1021/acsami.2c04341

[20] Didwischus N, Guduru A, Badylak SF, Modo M. In vitro dose-dependent effects of matrix metalloproteinases on ECM hydrogel biodegradation. Acta Biomater. 2024;174:104–115. 38081445 10.1016/j.actbio.2023.12.003 PMC10775082

[21] Deng J, Zhang X, Yin M, Cao W, Zhang B, Liu Q, Hou X, Wang H, Shi C. Modified CFBP-bFGF targeting to ischemic brain promoted the functional recovery of cerebral ischemia. J Control Release. 2023;353:462–474. 36493946 10.1016/j.jconrel.2022.12.007

[22] Li H, Gan X, Pan L, Zhang Y, Hu X, Wang Z. EGF/bFGF promotes survival, migration and differentiation into neurons of GFP-labeled rhesus monkey neural stem cells xenografted into the rat brain. Biochem Biophys Res Commun. 2022;620:76–82. 35780584 10.1016/j.bbrc.2022.06.077

[23] Wang ZG, Cheng Y, Yu XC, Ye LB, Xia QH, Johnson NR, Wei X, Chen DQ, Cao G, Fu XB, et al. bFGF protects against blood-brain barrier damage through junction protein regulation via PI3K-Akt-Rac1 pathway following traumatic brain injury. Mol Neurobiol. 2016;53(10):7298–7311. 26687235 10.1007/s12035-015-9583-6 PMC5516728

[24] Zhang R, Xie L, Wu F, Xu J, Lu L, Cao L, Li L, Meng W, Zhang H, Shao C, et al. ALG-bFGF hydrogel inhibiting autophagy contributes to protection of blood-spinal cord barrier integrity via PI3K/Akt/FOXO1/KLF4 pathway after SCI. Front Pharmacol. 2022;13: Article 828896. 35330841 10.3389/fphar.2022.828896 PMC8940228

[25] Ding Z, Dai C, Zhong L, Liu R, Gao W, Zhang H, Yin Z. Neuregulin-1 converts reactive astrocytes toward oligodendrocyte lineage cells via upregulating the PI3K-AKT-mTOR pathway to repair spinal cord injury. Biomed Pharmacother. 2021;134: Article 111168. 33395598 10.1016/j.biopha.2020.111168

[26] Cui MY, Fu YQ, Li ZL, Zheng Y, Yu Y, Zhang C, Zhang YQ, Gao BR, Chen WY, Lee YL, et al. Neuregulin-1/PI3K signaling effects on oligodendrocyte proliferation, remyelination and behaviors deficit in a male mouse model of ischemic stroke. Exp Neurol. 2023;362: Article 114323. 36690057 10.1016/j.expneurol.2023.114323

[27] Ma Q, Li R, Wang L, Yin P, Wang Y, Yan C, Ren Y, Qian Z, Vaughn MG, McMillin SE, et al. Temporal trend and attributable risk factors of stroke burden in China, 1990-2019: An analysis for the global burden of disease study 2019. Lancet Public Health. 2021;6(12):e897–e906. 34838196 10.1016/S2468-2667(21)00228-0 PMC9047702

[28] Yang Y, Gu B, Xu XY. In silico study of combination thrombolytic therapy with alteplase and mutant pro-urokinase for fibrinolysis in ischemic stroke. Comput Biol Med. 2024;171: Article 108141. 38367449 10.1016/j.compbiomed.2024.108141

[29] Nogueira RG, Pinheiro A, Brinjikji W, Abbasi M, Al-Bayati AR, Mohammaden MH, Viana LS, Ferreira F, Abdelhamid H, Bhatt NR, et al. Clot composition and recanalization outcomes in mechanical thrombectomy. J Neurointerv Surg. 2024;jnis-2023-020117. 10.1136/jnis-2023-020117 37419694

[30] Muraoka T, Ajioka I. Self-assembling molecular medicine for the subacute phase of ischemic stroke. Neurochem Res. 2022;47(9):2488–2498. 35666393 10.1007/s11064-022-03638-5 PMC9463329

[31] Dziki JL, Wang DS, Pineda C, Sicari BM, Rausch T, Badylak SF. Solubilized extracellular matrix bioscaffolds derived from diverse source tissues differentially influence macrophage phenotype. J Biomed Mater Res A. 2017;105(1):138–147. 27601305 10.1002/jbm.a.35894

[32] Massensini AR, Ghuman H, Saldin LT, Medberry CJ, Keane TJ, Nicholls FJ, Velankar SS, Badylak SF, Modo M. Concentration-dependent rheological properties of ECM hydrogel for intracerebral delivery to a stroke cavity. Acta Biomater. 2015;27:116–130. 26318805 10.1016/j.actbio.2015.08.040 PMC4609617

[33] Zhang M, Huang SS, He WY, Cao WJ, Sun MY, Zhu NW. Nasal administration of bFGF-loaded Nanoliposomes attenuates neuronal injury and cognitive deficits in mice with vascular dementia induced by repeated cerebral ischemia–reperfusion. Int J Nanomedicine. 2024;19:1431–1450. 38371455 10.2147/IJN.S452045 PMC10873211

[34] Wang C, Yang X, Jiang Y, Qi L, Zhuge D, Xu T, Guo Y, Deng M, Zhang W, Tian D, et al. Targeted delivery of fat extract by platelet membrane-cloaked nanocarriers for the treatment of ischemic stroke. J Nanobiotechnology. 2022;20(1):249. 35642036 10.1186/s12951-022-01461-2 PMC9153102

[35] Liao C, Guan Y, Zheng J, Wang X, Wang M, Zhu Z, Peng Q, Wang HH, Li M. Development of synthetic modulator enabling long-term propagation and neurogenesis of human embryonic stem cell-derived neural progenitor cells. Biol Res. 2023;56(1):59. 37951961 10.1186/s40659-023-00471-0 PMC10638775

[36] Jurcau A, Simion A. Neuroinflammation in cerebral ischemia and ischemia/reperfusion injuries: From pathophysiology to therapeutic strategies. Int J Mol Sci. 2021;23(1):14. 35008440 10.3390/ijms23010014 PMC8744548

[37] Wysocka A, Szczygielski J, Kopanska M, Oertel JM, Glowniak A. Matrix metalloproteinases in cardioembolic stroke: From background to complications. Int J Mol Sci. 2023;24(4):3628. 36835040 10.3390/ijms24043628 PMC9959608

[38] Shahriary GM, Kataria H, Karimi-Abdolrezaee S. Neuregulin-1 fosters supportive interactions between microglia and neural stem/progenitor cells. Stem Cells Int. 2019;2019:8397158. 31089334 10.1155/2019/8397158 PMC6476022

[39] Wang S, Li Y, Paudyal R, Ford BD, Zhang X. Evaluation of neuregulin-1’s neuroprotection against ischemic injury in rats using diffusion tensor imaging. Magn Reson Imaging. 2018;53:63–70. 30021123 10.1016/j.mri.2018.07.002

[40] Noll JM, Li Y, Distel TJ, Ford GD, Ford BD. Neuroprotection by exogenous and endogenous Neuregulin-1 in mouse models of focal ischemic stroke. J Mol Neurosci. 2019;69(2):333–342. 31290093 10.1007/s12031-019-01362-4

[41] Chambliss C, Stiles JK, Gee BE. Neuregulin-1 attenuates hemolysis- and ischemia induced-cerebrovascular inflammation associated with sickle cell disease. J Stroke Cerebrovasc Dis. 2023;32(2): Article 106912. 36473396 10.1016/j.jstrokecerebrovasdis.2022.106912 PMC10448832

[42] Simmons LJ, Surles-Zeigler MC, Li Y, Ford GD, Newman GD, Ford BD. Regulation of inflammatory responses by neuregulin-1 in brain ischemia and microglial cells in vitro involves the NF-kappa B pathway. J Neuroinflammation. 2016;13(1):237. 27596278 10.1186/s12974-016-0703-7 PMC5011915

[43] Gui C, Zeng ZY, Chen Q, Luo YW, Li L, Chen LL. Neuregulin-1 promotes myocardial angiogenesis in the rat model of diabetic cardiomyopathy. Cell Physiol Biochem. 2018;46(6):2325–2334. 29742506 10.1159/000489622

[44] Chen TT, Luque A, Lee S, Anderson SM, Segura T, Iruela-Arispe ML. Anchorage of VEGF to the extracellular matrix conveys differential signaling responses to endothelial cells. J Cell Biol. 2010;188(4):595–609. 20176926 10.1083/jcb.200906044 PMC2828913

