# Supplementary material for: Engineered Basic Fibroblast Growth Factor Specifically Bonded with Injectable Extracellular Matrix Hydrogel for the Functional Restoration of Cerebral Ischemia in Rats
Source: Biomater Res. 2024 May 2;28:0020. doi: 10.34133/bmr.0020 (PMC11075669; doi:10.34133/bmr.0020)
Supplement: Supplementary 1 — Fig. S1 [file bmr.0020.f1.zip › Supplemental Material-shi.docx]

Supplementary Information

Engineered basic fibroblast growth factor specifically bonded with injectable Extracellular matrix hydrogel for the functional restoration of cerebral ischemia in rats

Supplementary Fig 1


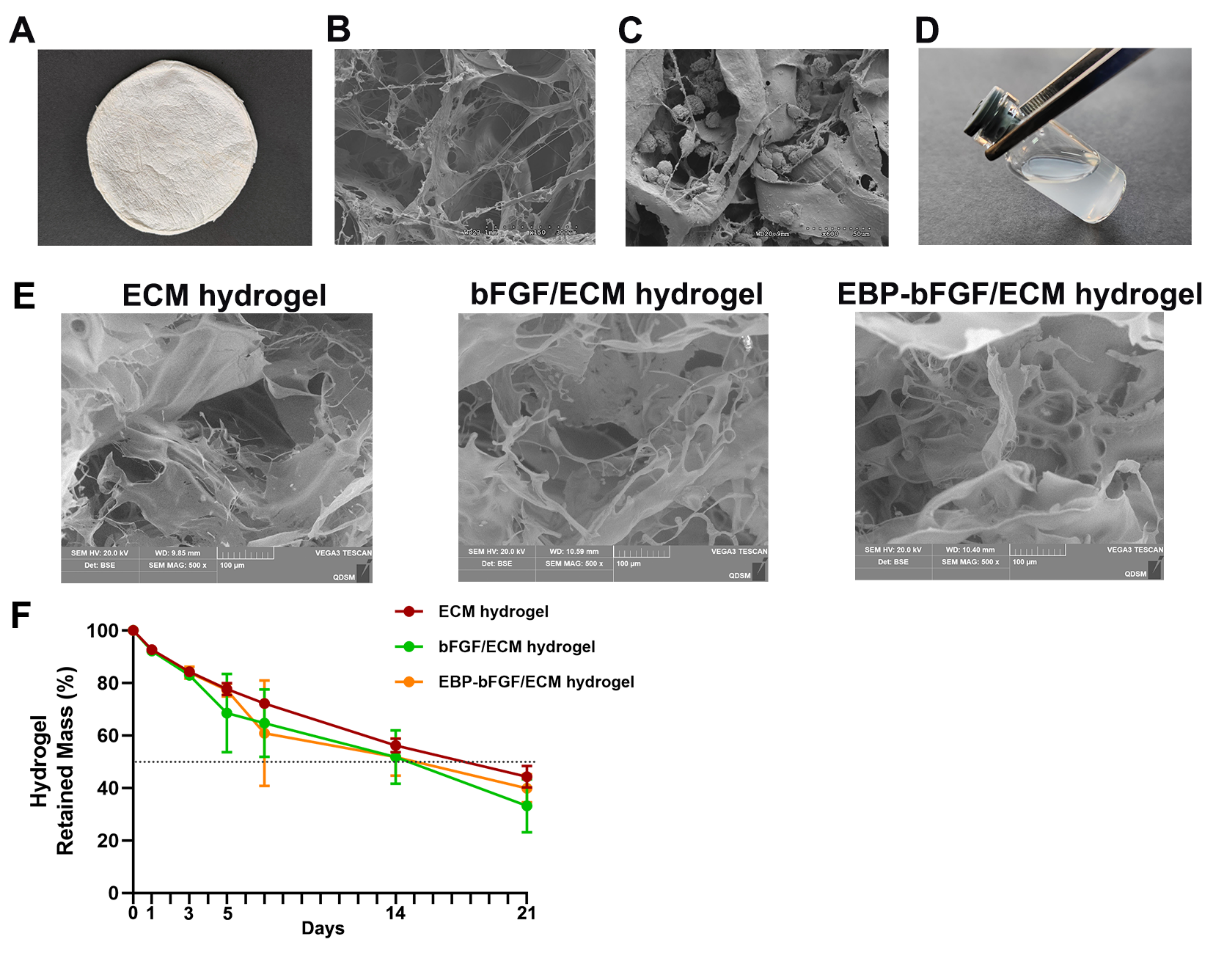


Supplementary Fig. 1: The preparation and characteristic of Extracellular matrix hydrogel. (A) Extracellular matrix stored at room temperature after drying. (B) Visual observation of Extracellular Matrix (ECM) hydrogel. (C-D) Porous structure and scaffold structure of ECM. ECM with an average pore size of 100 μm formed a 3-dimensional spatial structure for cell adhesion, growth, and mechanotransduction. (E) Images of ECM, bFGF/ECM and EBP-bFGF/ECM captured by scanning electron microscopy. Compared with ECM, the structure of ECM modified with bFGF and EBP-bFGF remained unchanged. (F) Degradation experiment of ECM, bFGF/ECM and EBP-bFGF/ECM hydrogels. ECM, bFGF/ECM and EBP-bFGF/ECM hydrogels were immersed in phosphate buffered saline (PBS) and eroded by PBS over 21 days. Record the mass change of hydrogels over 21 days.
