# Supplementary figures and images for: Engineered Basic Fibroblast Growth Factor Specifically Bonded with Injectable Extracellular Matrix Hydrogel for the Functional Restoration of Cerebral Ischemia in Rats
Source: Biomater Res. 2024 May 2;28:0020. doi: 10.34133/bmr.0020 (PMC11075669; doi:10.34133/bmr.0020)

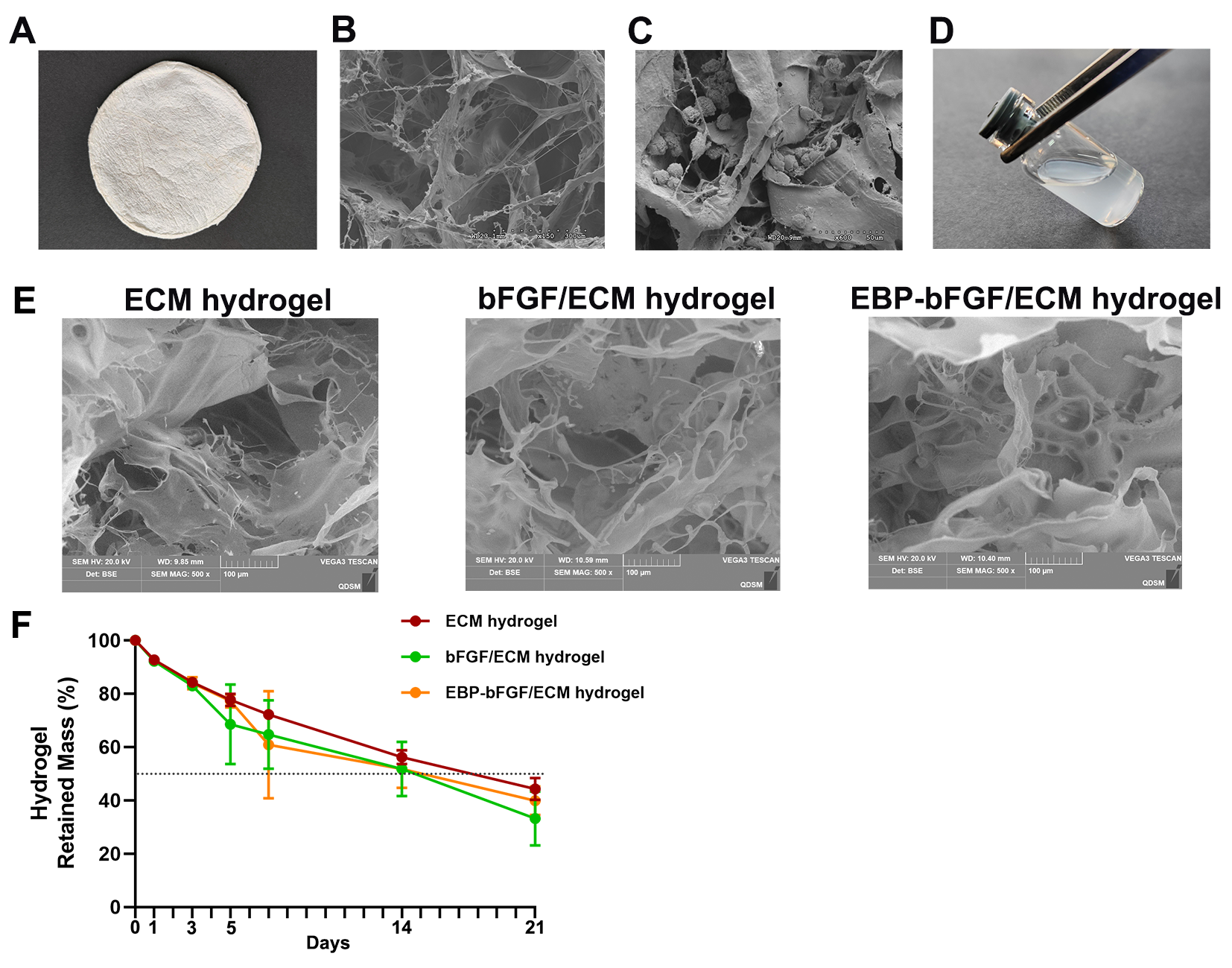

Supplement: Supplementary 1 — Fig. S1 [file bmr.0020.f1.zip › supplementary fig.tif]
